# A Mitochondrial Dysfunction and Oxidative Stress Pathway-Based Prognostic Signature for Clear Cell Renal Cell Carcinoma

**DOI:** 10.1155/2021/9939331

**Published:** 2021-11-24

**Authors:** Yue Wu, Xi Zhang, Xian Wei, Huan Feng, Bintao Hu, Zhiyao Deng, Bo Liu, Yang Luan, Yajun Ruan, Xiaming Liu, Zhuo Liu, Jihong Liu, Tao Wang

**Affiliations:** ^1^Department of Urology, Tongji Hospital, Tongji Medical College, Huazhong University of Science and Technology, Wuhan, 430030 Hubei, China; ^2^Institute of Urology, Tongji Hospital, Tongji Medical College, Huazhong University of Science and Technology, Wuhan, 430030 Hubei, China; ^3^The First Clinical Medical College of Anhui Medical University, Hefei, 230001 Anhui, China; ^4^Department of Oncology, Tongji Hospital, Tongji Medical College, Huazhong University of Science and Technology, Wuhan, 430030 Hubei, China

## Abstract

Mitochondria not only are the main source of ATP synthesis but also regulate cellular redox balance and calcium homeostasis. Its dysfunction can lead to a variety of diseases and promote cancer and metastasis. In this study, we aimed to explore the molecular characteristics and prognostic significance of mitochondrial genes (MTGs) related to oxidative stress in clear cell renal cell carcinoma (ccRCC). A total of 75 differentially expressed MTGs were analyzed from The Cancer Genome Atlas (TCGA) database, including 46 upregulated and 29 downregulated MTGs. Further analysis screened 6 prognostic-related MTGs (*ACAD11*, *ACADSB*, *BID*, *PYCR1*, *SLC25A27*, and *STAR*) and was used to develop a signature. Kaplan-Meier survival and receiver operating characteristic (ROC) curve analyses showed that the signature could accurately distinguish patients with poor prognosis and had good individual risk stratification and prognostic potential. Stratified analysis based on different clinical variables indicated that the signature could be used to evaluate tumor progression in ccRCC. Moreover, we found that there were significant differences in immune cell infiltration between the low- and high-risk groups based on the signature and that ccRCC patients in the low-risk group responded better to immunotherapy than those in the high-risk group (46.59% vs 35.34%, *P* = 0.008). We also found that the expression levels of these prognostic MTGs were significantly associated with drug sensitivity in multiple ccRCC cell lines. Our study for the first time elucidates the biological function and prognostic significance of mitochondrial molecules associated with oxidative stress and provides a new protocol for evaluating treatment strategies targeting mitochondria in ccRCC patients.

## 1. Introduction

Renal cell carcinoma (RCC) is a common and highly malignant tumor of the urinary system. Among them, clear cell renal cell carcinoma (ccRCC) accounts for 75–80% of all RCC [1, 2]. Symptoms of ccRCC are not obvious in the early stage, and about 25–30% of patients have metastases by the time of initial diagnosis [3]. In addition, metastatic recurrence occurred in approximately 30% of patients following nephrectomy [4]. CcRCC has a poor prognosis due to its high resistance to chemotherapy and radiotherapy [5], and the five-year survival rate for advanced ccRCC patients has been reported to be only 11.7% [6]. Although several prognostic factors for survival in patients with RCC have been described and signatures have been developed [7, 8], few signatures can be used clinically to predict prognosis in patients with RCC and reliable predictive biomarkers of individual sensitivity or drug resistance have not been identified. Moreover, in well-designed clinical trials, available evidence can only partially promote more precise and personalized patient selection [9]. Thus, in the era of a new generation of antiangiogenesis and immunotherapy, the need to cover unmet medical options may be critical.

Mitochondria is an important ancient organelle existing in eukaryotic cells. Its main function is to generate energy for cell survival through oxidative phosphorylation, maintain calcium homeostasis, and serve as a key component of cell apoptosis [10]. Reprogramming of cell metabolism and an abnormal redox status are considered to be the main characteristics of tumor transformation. So mitochondrial dysfunction plays an important role in a range of diseases, including diabetes, cancer, and neurodegenerative diseases [11]. The release of cytochrome C and the production of mitochondrial reactive oxygen species (mtROS) and metabolites due to mitochondrial dysfunction can initiate signaling cascades that affect gene expression and cell activation, proliferation, and differentiation [12, 13]. Importantly, the main source of intracellular reactive oxygen species (ROS) is the mitochondrial respiratory chain, which plays an important role in the maintaining redox balance and intracellular signal transduction [14]. Mitochondrial dysfunction increases intracellular oxidation and stress, destroys the functional activities of endoplasmic reticulum, lysosome, and other organelles, induces autophagy, and mediates cell damage and death. At the same time, the elevated ROS, as endogenous DNA destruction factors, promotes genetic instability, which may eventually lead to homeostasis and pathological disorders [13, 15]. Moreover, escalated ROS generation inhibits the activity of some key enzymes of energy metabolism such as NADH dehydrogenase, succinate dehydrogenase, and aconitase and causes mitochondrial DNA (mtDNA) damage and mutations [16]. Recently, Marquardt et al. [17] clustered RNA-sequencing data from three histopathological groups of RCC and revealed a unique histologically independent subgroup (mixed subgroup) characterized by enhanced mitochondria and weakened angiogenesis-related gene signatures. The association with the mixed subgroup significantly shortened the overall survival (OS) of patients with ccRCC and extended the overall survival of patients with chromophobe RCC (chRCC). Therefore, in-depth investigation of the molecular characteristics and biological functions of mitochondrial dysfunction and oxidative stress in ccRCC is helpful to further clarify the mechanism of tumor progression and identify reliable biomarkers.

Traditional research methods cannot reflect the molecular landscape of a large number of mitochondrial genes (MTGs). In addition, oncogenesis is a highly coordinated interaction of multiple regulatory factors, which requires a more comprehensive and effective analysis of the characteristics of MTGs in ccRCC. In this study, we explored the molecular characteristics and biological functions of MTGs related to oxidative stress in ccRCC by analyzing the genomic information from TCGA-KIRC. Besides, we developed a prognostic signature to predict ccRCC patient outcomes, explored its upstream regulatory mechanisms, and preliminarily revealed its potential to predict immunotherapy and drug sensitivity of ccRCC cell lines.

## 2. Materials and Methods

### 2.1. Data Collection and Differentially Expressed MTGs

Transcriptome data (read counts) of ccRCC patients and corresponding clinical data including the survival status, age, gender, grade, and stage were obtained from The Cancer Genome Atlas (TCGA, https://portal.gdc.cancer.gov/) database. A total of 539 ccRCC patients were enrolled in our study. The raw expression data was normalized by the trimmed mean of *M* values (TMM) algorithm in “edgeR” package, and the genes with average expression less than 1 were removed. The “edgeR” package was also used for MTG differential expression analysis. In our study, the criteria for screening differentially expressed MTGs were ∣log_2_ fold change (FC) | ≥1.2 and false discovery rate (FDR) < 0.05. The read counts were then converted to TPM values and performed a log_2_(*x* + 1) transformation for subsequent analysis, since the TPM values are the same as the microarray values [18]. Next, we downloaded 1136 MTGs from MitoCarta3.0 database (http://www.broadinstitute.org/mitocarta) [19], and according to the search term “oxidative stress,” 9469 human genes related to oxidative stress were obtained from OMIM database (https://www.oncomine.org/resource/), NCBI gene function module (https://www.ncbi.nlm.nih.gov/gene/), and GeneCard database (https://www.genecards.org/). Based on this, we screened a total of 788 MTGs related to oxidative stress for subsequent analysis. Moreover, the two expression matrices (E-MTAB-1980 and GSE29609 cohorts) and their corresponding clinical information were directly downloaded from the ArrayExpress database (https://www.ebi.ac.uk/arrayexpress/) and the Gene Expression Omnibus (GEO) database (http://www.ncbi.nlm.nih.gov/geo/), respectively, and used as external validation cohorts to validate the predictive performance of the signature. Additionally, we downloaded transcriptome data of 65 patients with chRCC including OS, disease-free survival (DFS), disease-specific survival (DSS), progression-free survival (PFS), and other clinical information from the cBioPortal database (https://www.cbioportal.org/datasets).

### 2.2. Weighted Correlation Network Analysis (WGCNA)

WGCNA analysis is an important method to identify key genes and evaluate the relationship between key modules. To assess the relationship between differentially expressed MTGs and clinical variables as a whole, we performed WGCNA on differentially expressed MTGs using the R “WGCNA” package. A soft threshold was set to obtain the optimal scale-free topology fitting model index (scale-free *R*^2^), and the degree of difference between genes was determined based on the topological overlap metric. After clustering the modules and genes, the correlation between the clinical variables in TCGA and the module characteristic genes was analyzed. *P* < 0.05 was considered statistically significant.

### 2.3. Construction and Validation of a MTG-Based Prognostic Signature

We performed univariate, least absolute shrinkage and selection operator (LASSO), and multiple stepwise Cox regression analyses sequentially in these 539 ccRCC patients to identify the MTGs most associated with prognosis. The risk score for predicting the prognostic risk in ccRCC patients was then calculated with the following formula:

(1) $$\begin{array}{c} \text{Risk score}=\overset{n}{\underset{i=1}{\sum }}\text{E}\text{xp}i\beta i. \end{array}$$

In the above formula, Exp and *β* represent the normalized expression value and regression coefficient of gene *i*, respectively. Next, all ccRCC patients were grouped according to the median risk score (high- and low-risk groups). Kaplan-Meier analysis was used to compare the difference in OS between the two groups. The receiver operating characteristic (ROC) curves were used to evaluate the accuracy and diagnostic value of the MTG-based prognostic signature. Moreover, the E-MTAB-1980 and GSE29609 cohorts were used as external cohorts to further evaluate the signature.

### 2.4. Exploration of the Relationship between the Prognostic Signature, Prognostic MTGs, and Clinical Variables

The differences in prognosis and the distribution of risk scores were explored under different clinical variable stratification. In addition, we further analyzed the correlation between these prognostic MTGs and different clinical variables to reveal their possible roles in ccRCC.

### 2.5. Regulatory Network of Transcription Factors (TFs) and MTGs and Functional Enrichment Analysis

We constructed a TF-MTG regulatory network based on coexpression analysis to reveal the potential functions of TFs and MTGs in ccRCC. We identified 318 transcription factors associated with tumorigenesis and progression from cistrome (https://cistrome.org/) [20]. Next, the overlap was extracted from the expression data of TCGA and analyzed for differential expression. Then, we carried out coexpression analysis on these differentially expressed TFs and prognostic MTGs based on ∣Cor | >0.3 and *P* < 0.001 standard. Besides, we explored the Gene Ontology (GO) and Kyoto Encyclopedia of Genes and Genomes (KEGG) database pathways based on the “clusterProfiler” package to clarify the molecular functions and key signaling pathways of differentially expressed MTGs.

### 2.6. Establish and Evaluate a Nomogram

We first performed univariate and multivariate Cox regression analyses for the risk score and clinical variables. Next, based on the R “rms” package, a nomogram combining clinical variables and MTG-based risk signature was constructed. Kaplan-Meier survival curves and ROC curves were performed in TCGA and E-MTAB-1980 cohorts, respectively, to evaluate the performance of the nomogram.

### 2.7. Assessment of Immune Cell Infiltration and Immunotherapy

Cell type identification by estimating relative subsets of RNA transcripts (CIBERSORT) is a deconvolution algorithm developed by Newman et al. [21], which is primarily used to calculate the abundance of infiltrated immune cells per sample. We estimated the degree of immune cell infiltration in different risk groups based on CIBERSORT and its gene set LM22. The algorithm was simulated 1000 times, and *P* < 0.05 was the screening criteria. Next, the tumor immune dysfunction and exclusion (TIDE) was developed by Jiang et al. [22] to predict the response to immunotherapy based on the simulation of the tumor immune escape mechanism. In this paper, the response of the TCGA-KIRC cohort to immunotherapy was preliminarily discussed based on TIDE algorithm, due to the lack of open-access data of the ccRCC cohort receiving immunotherapy.

### 2.8. Immunofluorescence Assay

The ccRCC and normal renal tissue were immobilized in 10% phosphate formalin solution, then paraffin embedded, and sectioned to 5 *μ*m thickness. The paraffin sections were then dewaxed and antigen repaired. Next, the sections were closed with goat serum at room temperature for 30 min and incubated overnight with CD163 antibody (1 : 200) at 4°C (Abcam, Cambridge, MA, USA). The sections were then incubated in CY3-labeled fluorescent secondary antibody for 1 h and DAPI dye for 10 min. Finally, the images were observed and collected under a fluorescence microscope (Nikon Eclipse C1, Nikon, Tokyo, Japan).

### 2.9. Relationship between the Prognostic MTG Expression Level and Drug Sensitivity of Various ccRCC Cell Lines

We downloaded the genomic expression data of tumor cell lines and the corresponding drug therapy IC50 from the GDSC database (https://www.cancerrxgene.org/) to analyze the relationship between the expression level of prognostic MTGs and IC50 values of various targeted drugs in ccRCC cell lines.

### 2.10. Cell Transfection and Real-Time Quantitative Polymerase Chain Reaction (RT-qPCR)

A PBX1 expression plasmid and small interfering RNA (siRNA) targeting PBX1 (PBX1 siRNA) were used for gain- and loss-of-function analyses, respectively. The PBX1 expression plasmid, PBX1 siRNA, and their negative control (NC) plasmid were synthesized by GeneChem (Shanghai, China). OS cells were seeded in a 6-well plate at 5 × 105 cells per well and transfected according to the manufacturer's instructions (Invitrogen).

Total RNA was extracted from ccRCC tumor tissues and normal kidney tissues with TRIzol reagent (Beyotime, Jiangsu, China), and the reverse transcriptional reaction was performed using the PrimeScript™ RT Reagent Kit (perfect real time) (TaKaRa, Japan). RT-qPCR was then performed in the ABI Prism 7300 system (Thermo Fisher Scientific) using TB Green® Premix Ex Taq™ II (Tli RNaseH Plus) (TaKaRa, Japan) according to the manufacturer's instructions. The relevant primer data are shown in Table S1. The fold change value of mRNA was calculated by the 2^−∆∆*Ct*^ method.

### 2.11. Statistical Analysis

In this study, R version 4.0.5 and GraphPad Prism 8.0 were used for all the calculations and statistical analysis. The “edgeR” package was used for normalization and differential analysis of the expressed data. The MTGs with ∣log2 FC | ≥1.2 and FDR < 0.05 (adjusted *P* < 0.05) were defined as differentially expressed genes. We first converted the FDR value of each gene to −log10 and then generated the volcano plot using the “ggplot2,” “dplyr,” and “ggrepel” packages. The “pheatmap” package was used to generate heatmaps. Univariate and multivariate Cox regression analysis was performed in the “survival” package, and LASSO regression analysis was performed in the “glmnet” package to identify MTGs associated with OS. The Kaplan-Meier method in the “survival” package was used for survival curve analysis, and the differences between groups were tested by log-rank test. The “ggplot2” package was used to generate ROC curves. The risk curve and survival status plot of each patient were generated by R “pheatmap” package. The “ggplot2” and “ggpubr” packages were used to generate boxplots and violin plots. The coexpression of differentially expressed TFs and prognostic MTGs was analyzed by Pearson correlation test in R “cor.test” function. Meeting the ∣Cor | >0.3 and *P* < 0.001 criteria indicated an association. According to the results of coexpression analysis, we used R “ggplot2,” “ggalluvial,” and “RColorBrewer” packages to generate the Sankey plot. The “clusterProfiler” package was used for GO and KEGG enrichment analysis. The R “rms” package was used to construct a nomogram to quantitatively predict the outcome of patients with ccRCC. The encapsulated CIBERSORT.R script and LM22 gene set are available from the CIBERSORT (https://cibersort.stanford.edu/download.php) website. Then, we evaluated the degree of immune cell infiltration in each patient based on R software. Pearson correlation analysis was used to calculate the correlation between drug IC50 and prognostic MTG expression values. ∣Rs | >0.2 and *P* < 0.05 were considered to be correlated. The unpaired Student's *t*-test was used to compare two groups of normally distributed variables, while the Mann–Whitney *U* test was used to compare two groups of non-normally distributed variables. The variables in the contingency table were analyzed by the chi-square tests or Fisher's exact tests.

## 3. Results

### 3.1. Differential Expression MTG Analysis

**Figure 1:**
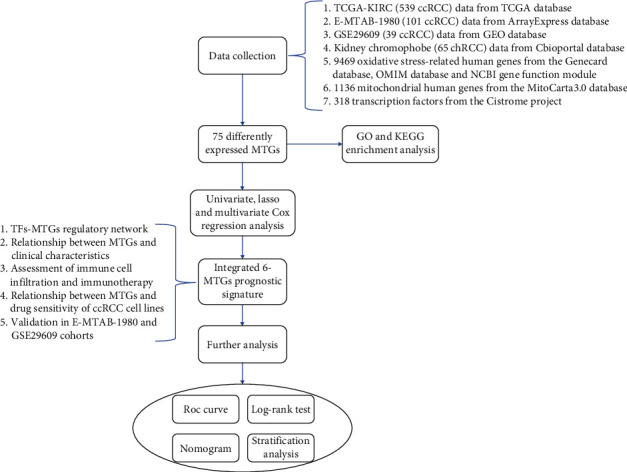
Workflow of the study. We developed a MTG-based prognostic signature associated with oxidative stress based on the TCGA-KIRC cohort and validated it in the E-MTAB-1980 and GSE29609 cohorts.

**Figure 2:**
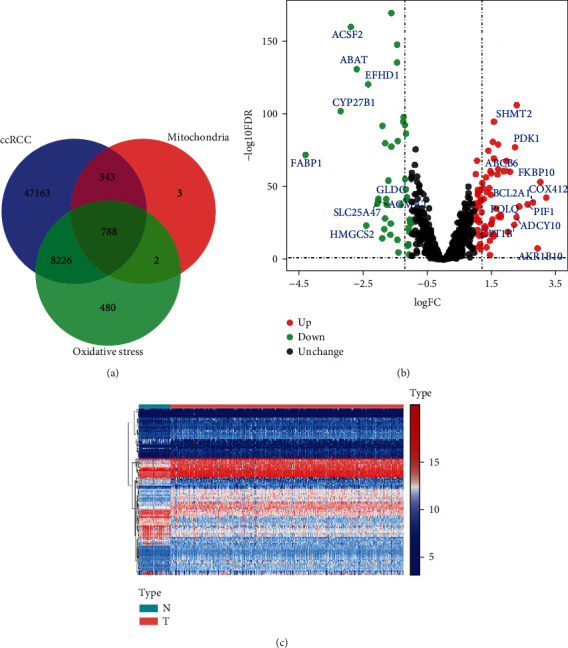
Analysis of mitochondrial genes associated with oxidative stress. (a) Venn diagram depicting intersecting genes in ccRCC/mitochondria/oxidative stress; (b) volcano plot of differentially expressed MTGs; (c) heatmap of differentially expressed MTGs. N represented the normal group and T represented the tumor group. (The statistical method was multiple hypothesis testing; the reported *P* value was the fdr value).

Figure 1 shows the workflow of this study. After preliminary screening, a total of 788 genes related to oxidative stress were identified.  Figure 2(a) shows the Venn diagram of screening. Subsequently, a total of 75 differentially expressed MTGs were identified in ccRCC and normal kidney tissues, including 46 upregulated MTGs and 29 downregulated MTGs. The log_2_FC values of all differentially expressed MTGs in ccRCC patients and the corresponding −log_10_FDR values were shown in Figure 2(b), and the expression levels of all differentially expressed MTGs were shown in Figure 2(c). Additionally, after WGCNA analysis, the relationship between the module and different clinical variables, including the age, gender, tumor grade, tumor stage, T stage, M stage, and N stage, is shown in Figure S1. The results showed that the two modules were negatively correlated with the tumor stage, T stage and M stage, respectively (*P* < 0.01). One module was negatively correlated with the N stage (*P* < 0.05). However, there was no significant correlation between the gene modules and the age, gender, or tumor grade. The above results indicated that the 75 differentially expressed MTGs were worthy of subsequent analysis.

### 3.2. Construction and Validation of a MTG-Based Prognostic Signature

**Figure 3:**
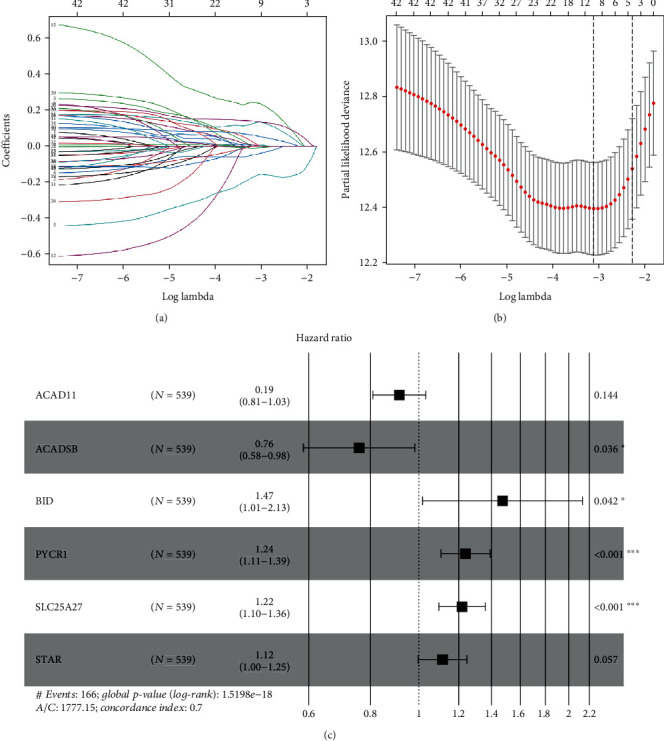
Screening of MTGs related to prognosis in patients with ccRCC. (a) The trajectory changes of the 62 independent variable coefficients; (b) the crossvalidation results of model construction; (c) multivariate Cox regression analysis of 9 MTGs. (The statistical method was multiple hypothesis testing; the reported *P* value was the fdr value).

We identified 62 prognostic-related MTGs by univariate Cox regression analysis (Table S2). Then, LASSO regression analysis was performed on these MTGs based on the “glmnet” package to screen out the MTGs closely related to OS and a total of 9 MTGs were identified, including *ACAD11*, *ACADSB*, *ATAD3B*, *BID*, *FKBP10*, *HMGCS2*, *PYCR1*, *SLC25A27*, and *STAR*. The trajectory changes of the 62 independent variable coefficients and the crossvalidation results of model construction are shown in Figures 3(a) and 3(b). Afterwards, we performed multiple stepwise Cox regression analysis on these 9 MTGs and further screened out 6 MTGs that were most relevant to the prognosis of patients with ccRCC based on AIC information statistics, including *ACAD11*, *ACADSB*, *BID*, *PYCR1*, *SLC25A27*, and *STAR* (Figure 3(c)). We then constructed a prognostic signature based on the *β* coefficients obtained from multivariate Cox regression analysis and corresponding gene expression values. The specific calculation formula of the risk score was shown below:

(2) $$\begin{array}{c} \text{Risk score}=\left(-0.0911\times \text{ExpACAD}11\right)+\left(-0.2774\times \text{ExpACADSB}\right)+\left(0.3854\times \text{ExpBID}\right)+\left(0.2156\times \text{ExpPYCR}1\right)+\left(0.2005\times \text{ExpSLC}25\text{A}27\right)+\left(0.1091\times \text{ExpSTAR}\right). \end{array}$$

**Figure 4:**
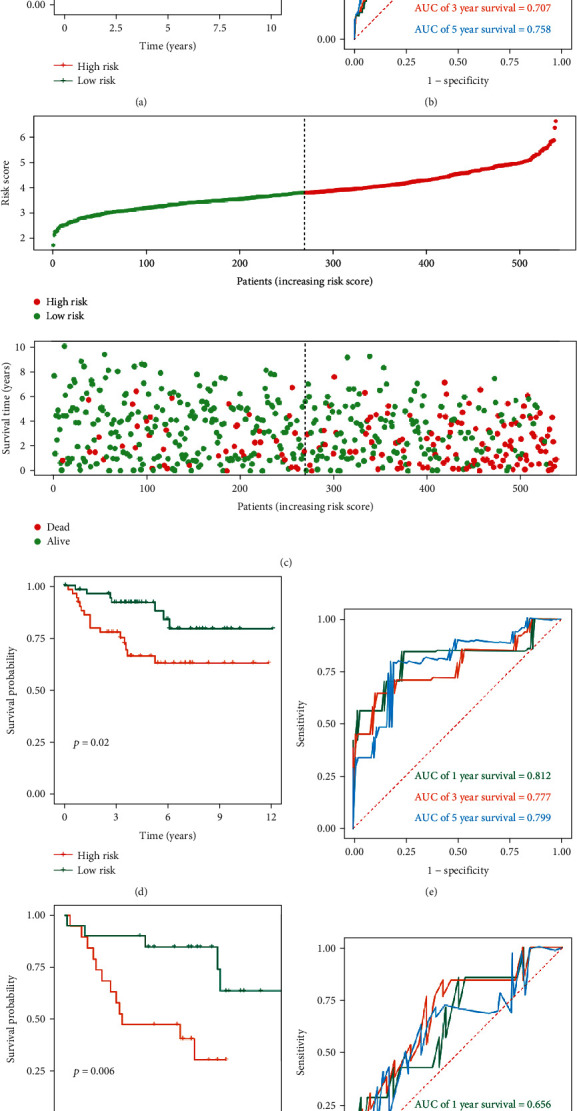
Prognostic value of MTG-based signature based on TCGA, E-MTAB-1980, and GSE29609 cohorts. (a) Kaplan-Meier survival curve analysis for overall survival grouped by the median risk score in the TCGA cohort; (b) time-dependent ROC curves measuring the predictive value of the risk score in the TCGA cohort; (c) distribution of the risk score, overall survival, and survival status of the prognostic signature in the TCGA cohort; (d) Kaplan-Meier survival curve analysis for overall survival grouped by the median risk score in the E-MTAB-1980 cohort; (e) time-dependent ROC curves measuring the predictive value of the risk score in the E-MTAB-1980 cohort; (f) Kaplan-Meier survival curve analysis for overall survival grouped by the median risk score in the GSE29609 cohort; (g) time-dependent ROC curves measuring the predictive value of the risk score in the GSE29609 cohort. (The statistical method was a log-rank test for a single factor, with only one test and the reported *P* value was the *P* value).

Then, 539 ccRCC patients in the TCGA cohort were divided into high-risk and low-risk groups based on the median risk score. The common clinical characteristics (age, gender, grade, stage, T stage, N stage, M stage, and survival status) between the high- and low-risk groups were shown in Table S3. The Kaplan-Meier method was used to analyze the OS of patients in the two groups, and the results showed that patients in the high-risk group had significantly shorter OS (*P* = 3.374*e* − 13, Figure 4(a)). Based on the ROC curve analysis, we evaluated the predictive performance and accuracy of the prognostic signature at one, three, and five years. The predicted area under the ROC curve (AUC) values for the signature at one, three, and five years were 0.736, 0.707, and 0.758, respectively (Figure 4(b)). The risk score, OS, and survival status distribution of ccRCC patients are shown in Figure 4(c). Next, we evaluated the stability and applicability of the prognostic signature according to the E-MTAB-1980 and GSE29609 external cohorts. The above formula was used to calculate the risk score for each patient in both cohorts. Then, Kaplan-Meier survival analysis showed consistent results with those described above (*P* = 0.020 and *P* = 0.006, Figures 4(d) and 4(f)). Based on the E-MTAB-1980 cohort, the predicted AUC values for the signature at one, three, and five years were 0.812, 0.777, and 0.799, respectively (Figure 4(e)). And based on the GSE29609 cohort, the predicted AUC values for the signature at one, three, and five years were 0.656, 0.699, and 0.639, respectively (Figure 4(g)). Therefore, we have sufficient evidence to show that the MTG-based prognostic signature has good stability and predictive performance.

### 3.3. Prognostic Significance of the Signature Stratified according to Common Clinical Variables

**Figure 5:**
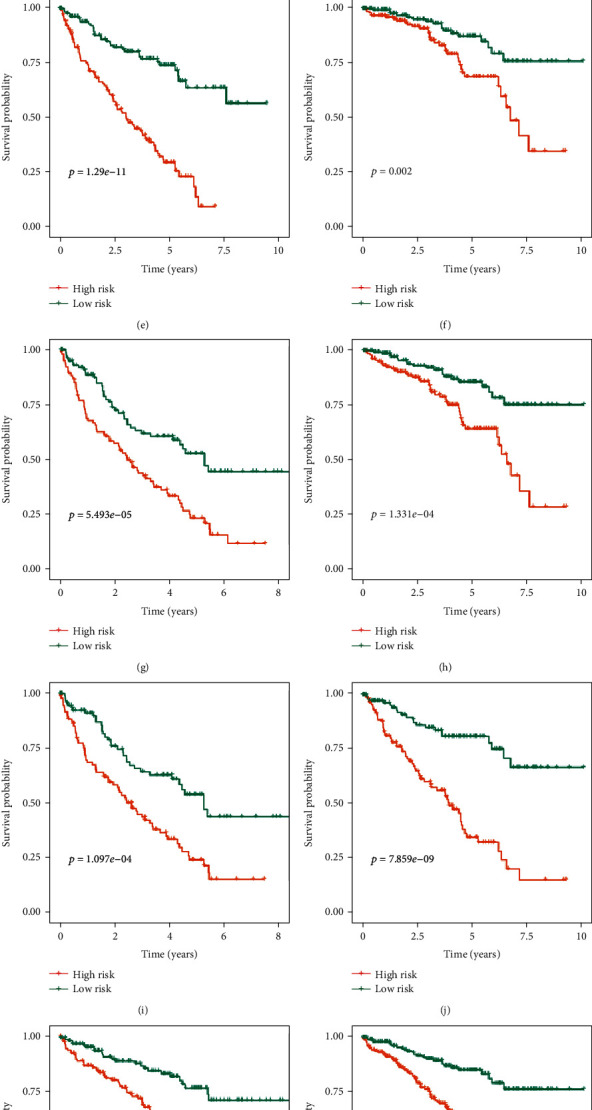
Kaplan-Meier survival curve analysis for overall survival stratified by different clinical variables. (a) Age ≤ 65; (b) age > 65; (c) male; (d) female; (e) grades 3–4; (f) stages I–II; (g) stages III–IV; (h) T stages 1–2; (i) T stages 3–4; (j) N stage 0; (k) N stages I–X; (l) M stage 0; (m) M stages I–X. (The statistical method was a log-rank test for a single factor, with only one test and the reported *P* value was the *P* value).

These 539 ccRCC patients were stratified according to different clinical variables to explore the signature's ability to identify patients with poor prognosis. The Kaplan-Meier method was used to analyze the survival of ccRCC patients in the high- and low-risk groups under different clinical stratification, and the results showed that the high-risk group had worse prognosis under different clinical variable stratification (Figure 5). These results show that the MTG-based prognostic signature can accurately screen out patients with poor prognosis without considering multiple clinical variables.

### 3.4. Relationship between the Prognostic Risk Score and Different Clinical Variables

**Figure 6:**
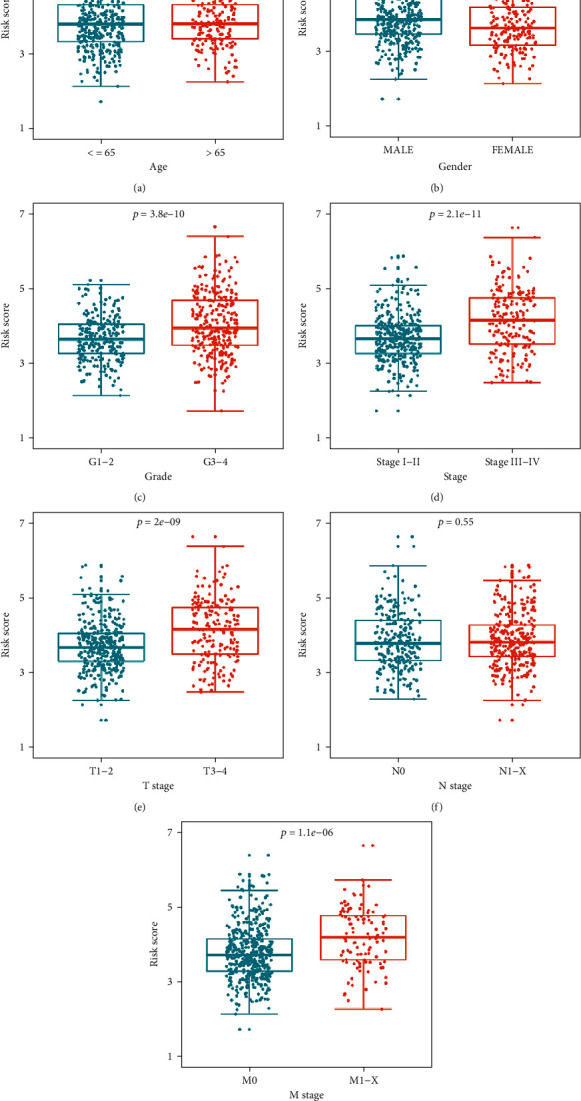
Distribution of the prognostic risk score under stratification of different clinical variables. (a) Age; (b) gender; (c) grade; (d) stage; (e) T stage; (f) N stage; (g) M stage. (Subgraphs (a), (b), (e), (f), and (g) were the *t*-test with only one test; subgraphs (c) and (d) were the nonparametric Mann–Whitney rank sum test with only one test. The reported *P* value was the fdr value).

Next, we analyzed the distribution of the prognostic risk score across different clinical variables to determine whether they were associated with disease progression. The results showed that the distribution of risk scores was not significantly different by age and N stage (Figures 6(a) and 6(f)). However, the distribution of risk scores differed significantly across other clinical variables. Specifically, it was higher in male patients than female patients (Figure 6(b)), it was higher in grade 3–4 than grade 1–2 (Figure 6(c)), it was higher in stage III–IV than stage I–II (Figure 6(d)), it was higher in T stage 3–4 than T stage 1–2 (Figure 6(e)), and it was higher in M stage I–X than M stage 0 (Figure 6(g)). The above results suggest that a higher prognostic risk score in ccRCC patients may be indicative of a higher degree of malignancy.

### 3.5. Relationship between Prognostic MTGs and Different Clinical Variables

We also further analyzed the relationships between these 6 prognostic MTGs and different clinical variables to understand their possible roles in ccRCC. The results indicated that there was significant correlation between ACAD11, BID, and PYCR1 and gender; ACAD11, ACADSB, BID, PYCR1, SLC25A27, and STAR were significantly correlated with the grade, stage, and T stage. ACAD11, ACADSB, BID, PYCR1, and STAR were significantly correlated with the M stage. However, no gene was significantly associated with the N stage (Table 1).

### 3.6. Assessment of the Efficacy of Prognostic MTGs and the Prognostic Signature

In order to evaluate the ability of these six prognostic MTGs to distinguish between ccRCC tumor tissue and normal tissue, as well as the ability of the signature to distinguish between ccRCC tumor malignancy, we performed ROC curve analysis. The results showed that the AUC of these six prognostic MTGs was ACAD11 (AUC = 0.669, *P* < 0.001), ACADSB (AUC = 0.943, *P* < 0.001), BID (AUC = 0.897, *P* < 0.001), PYCR1 (AUC = 0.710, *P* < 0.001), SLC25A27 (AUC = 0.591, *P* = 0.012), and STAR (AUC = 0.607, *P* = 0.003), which showed good diagnostic accuracy for ccRCC (Figure S2). Moreover, we also evaluated the ability of the prognostic signature to distinguish ccRCC tumor progression through the ROC curve and the AUC. The results showed that the AUC was 0.640 (95% CI = 0.593–0.687, *P* < 0.001) for the prediction of the tumor grade, the AUC was 0.669 (95% CI = 0.620–0.719, *P* < 0.001) for the prediction of the tumor stage, the AUC was 0.655 (95% CI = 0.604–0.706, *P* < 0.001) for the prediction of the T stage, and the AUC was 0.662 (95% CI = 0.603–0.721, *P* < 0.001) (Figure S2) for prediction of the M stage.

### 3.7. Gene Set Enrichment Analysis (GSEA) of Prognostic MTGs

In order to explore the potential functions of these six prognostic MTGs in ccRCC, we studied the genes mostly related to the expression of these six MTGs based on GSEA analysis to reflect their possible functions. The results showed that ACAD11 was mainly enriched in the FoxO signaling pathway, HIF-1 signaling pathway, RNA transport, Th1 and Th2 cell differentiation, TNF signaling pathway, and VEGF signaling pathway. ACADSB was mainly enriched in the citrate cycle (TCA cycle), glycolysis/gluconeogenesis, mTOR signaling pathway, peroxisome, PPAR signaling pathway, and Wnt signaling pathway. BID was mainly enriched in DNA replication, NF-kappa B signaling pathway, NOD-like receptor signaling pathway, p53 signaling pathway, proteasome, and ribosome. PYCR1 was mainly enriched in the cell cycle, Hippo signaling pathway, mRNA surveillance pathway, nucleotide excision repair, proteasome, and TGF-beta signaling pathway. SLC25A27 was mainly enriched in the glycerophospholipid metabolism, Notch signaling pathway, phosphatidylinositol signaling system, phospholipase D signaling pathway, spliceosome, and Th1 and Th2 cell differentiation. STAR was mainly enriched in the arachidonic acid metabolism, beta-alanine metabolism, calcium signaling pathway, citrate cycle (TCA cycle), fat digestion and absorption, and propanoate metabolism (Figure S3). These results suggested that these six prognostic MTGs may play an important role by affecting the redox homeostasis, energy production, immune infiltration, and metastasis mechanism of ccRCC.

### 3.8. Correlation of Prognostic MTGs with Immune Infiltration and RNA Modification in ccRCC

Considering that immunity plays a key role in a variety of diseases and the above results suggested that these six MTGs may affect the immune status of ccRCC, we investigated the relationship between the expression of these six MTGs and the level of immune infiltration of ccRCC. The results were shown in Figure S4. The expression level of ACAD11 was positively correlated with tumor purity and the infiltration levels of CD8+ T cells and B cells, but not with the infiltration levels of CD4+ T cells, dendritic cells, macrophages, and neutrophil. The expression level of ACADSB was positively correlated with tumor purity and the infiltration levels of CD4+ T cells, dendritic cells, and neutrophil, but not with the infiltration levels of CD8+ T cells, B cells, and dendritic cells. The expression level of BID was negatively correlated with tumor purity and the infiltration levels of B cells and has significant positive correlations with CD8+ T cells, dendritic cells, macrophages, and neutrophil, but not with the infiltration levels of CD4+ T cells. The expression level of PYCR1 was negatively correlated with tumor purity and has significant positive correlations with dendritic cells, but not with the infiltration levels of CD8+ T cells, CD4+ T cells, B cells, macrophages, and neutrophil. The expression level of SLC25A27 was positively correlated with tumor purity and the infiltration levels of CD4+ T cells, but not with the infiltration levels of CD8+ T cells, dendritic cells, B cells, macrophages, and neutrophil. The expression level of STAR was positively correlated with the infiltration levels of CD4+ T cells, but not with tumor purity and the infiltration levels of CD8+ T cells, dendritic cells, B cells, macrophages, and neutrophil. In addition, since RNA modification plays an important role in a variety of diseases, including tumors, and N6-methyladenosine (m6A) is the most abundant and characteristic modification in eukaryotic mRNA [23], we further studied the relationship between m6A modification enzyme (writers, erasers, and readers) and these six prognostic MTGs through Pearson analysis. The heatmap of the correlation matrix showed that the expression of ACAD11, ACADSB, BID, PYCR1 and SLC25A27 was significantly positively correlated with m6A modification (Figure S5).

### 3.9. Regulatory Network of TFs-MTGs and Functional Enrichment Analysis

**Figure 7:**
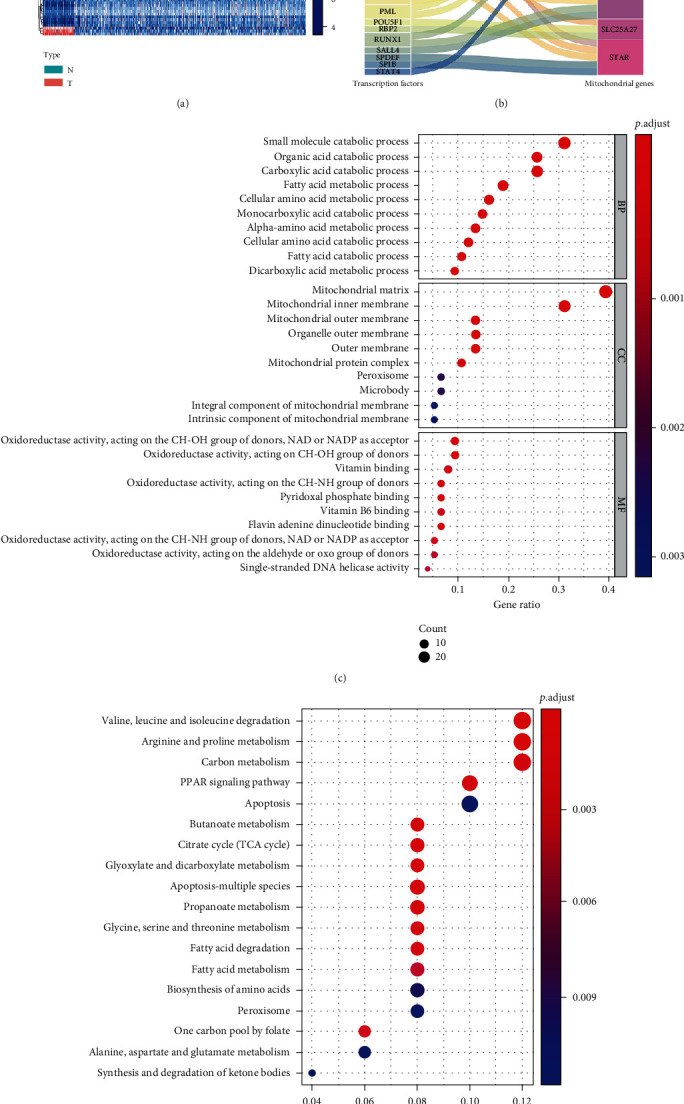
Regulatory network of TFs-MTGs and functional enrichment analysis. (a) Heatmap of differentially expressed TFs. N represented the normal group and T represented the tumor group; (b) Sankey plot of the TF-MTG regulatory network; (c) GO enrichment analysis of the differentially expressed MTGs; (d) KEGG enrichment analysis of the differentially expressed MTGs. (The statistical method was multiple hypothesis testing; the reported *P* value was the fdr value).

Considering that mitochondria not only produce energy for cell metabolic homeostasis and cell survival through oxidative phosphorylation but also participate in a variety of biological processes, including calcium homeostasis and signal transduction [24], thus, maintaining the mitochondrial number, morphology, and function, known as mitochondrial quality control (MQC), is essential for mitochondrial and cellular health. TFs play an important role in this process. Wang et al. [25] concluded that transcription factor EB (TFEB) plays a key role in MQC, mainly through activation of mitochondrial autophagy, regulation of mitochondrial biogenesis, removal of reactive oxygen species, and balance of the mitochondrial fission-fusion cycle, and therapeutic strategies targeting TFEB have certain therapeutic effects on diseases related to mitochondrial dysfunction. Another study revealed that Forkhead box O (FOXO) protects mitochondria by activating mitochondrial antioxidant enzymes and repairs or remodels damaged mitochondria by inducing mitochondrial autophagy to maintain cell and organism homeostasis [26]. Ryoo et al. [27] also found that nuclear factor, erythroid 2-like 2 (Nrf2) can be positively correlated with mitochondrial biology by directly upregulating mitochondrial transcription factors and participate in the MQC system by activating mitochondrial autophagy. Based on this, it is of great significance to fully reveal the regulatory network of TFs-MTGs. After final screening, 66 differentially expressed TFs were identified, including 46 upregulated and 20 downregulated TFs. The expression levels of all differentially expressed TFs were shown in Figure 7(a). Then, the regulatory network of TFs-MTGs was revealed by coexpression analysis (Figure 7(b)). A total of 26 TFs involved in upstream regulation were identified. The regulatory information between these TFs and prognostic MTGs was shown in Table S4. To further verify the regulatory relationship between these prognostic MTGs and TFs, we used the JASPAR online database (http://jaspar.genereg.net/) to predict the binding sites of these TFs in the promoter regions of these six prognostic MTGs. The sequence of predicted sites was shown in Table S5. Moreover, we also constructed overexpression and interference plasmids to further verify the regulation of TFs on these prognostic MTGs. The results were shown in Figure S6.

Additionally, further GO and KEGG enrichment analyses were performed to reveal the molecular functions and key signaling pathways of these differential MTGs. Biological process analysis showed that these MTGs were mainly concentrated in the small molecule catabolic process, organic acid catabolic process, cellular amino acid metabolic process, fatty acid catabolic process, and apoptotic mitochondrial changes. Cellular component analysis showed that these MTGs were mainly concentrated in the mitochondrial matrix, mitochondrial inner membrane, mitochondrial outer membrane, and mitochondrial nucleoid. Molecular function analysis showed that these MTGs were mainly concentrated in coenzyme binding, oxidoreductase activity, acting on the CH-NH group of donors, NADP-retinol dehydrogenase activity, NAD or NADP as an acceptor, DNA-dependent ATPase activity, electron transfer activity, and pyridoxal phosphate binding (Figure 7(c)). In terms of KEGG analysis, these differentially expressed MTGs were mainly concentrated in apoptosis-multiple species, citrate cycle, peroxisome, PPAR signaling pathway, biosynthesis of amino acids, and metabolism of various fatty acids and amino acids (Figure 7(d)). Moreover, we also performed functional enrichment analysis on these TFs involved in regulation, and the results showed that these TFs were mainly enriched in immune cell differentiation, immune cell infiltration, and immune response activation (Figure S7).

### 3.10. Establish and Evaluate a Nomogram

**Figure 8:**
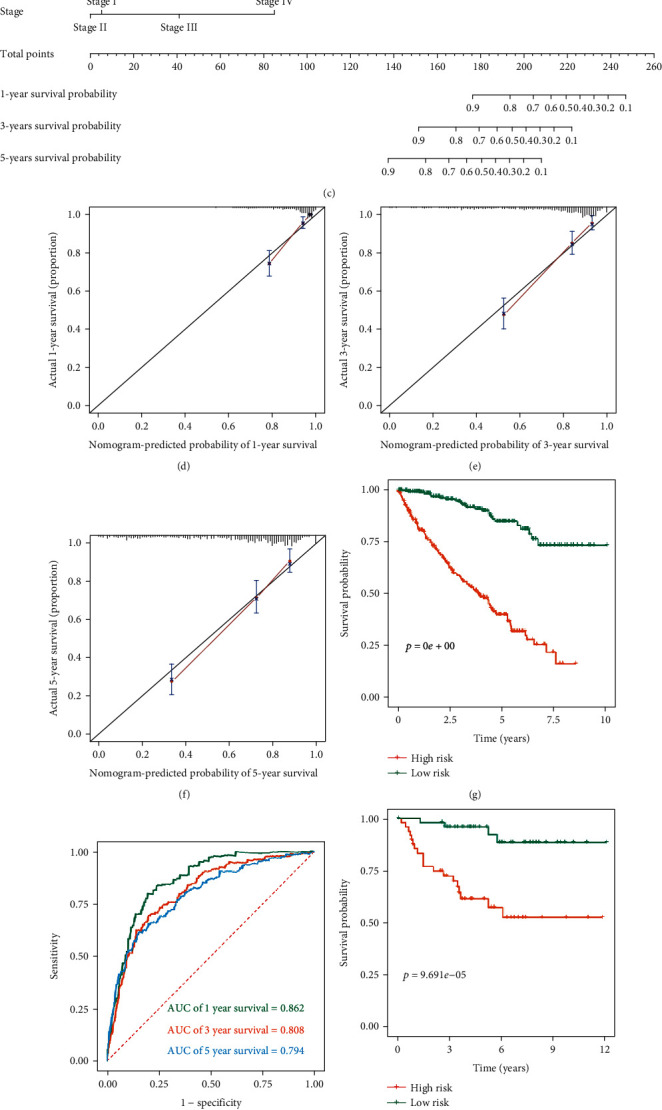
Establish and evaluate a nomogram. (a) Univariate Cox regression analysis for the risk score and clinical variables in the TCGA cohort; (b) multivariate Cox regression analysis for the risk score and clinical variables in the TCGA cohort; (c) the nomogram for predicting ccRCC patients at 1, 3, and 5 years; (d–f) calibration curves at 1, 3, and 5 years for the nomogram based on the TCGA cohort; (g) Kaplan-Meier survival curve analysis for overall survival grouped by the median risk score in the TCGA cohort based on the nomogram; (h) time-dependent ROC curves measuring the predictive value of the risk score in the TCGA cohort based on the nomogram; (i) Kaplan-Meier survival curve analysis for overall survival grouped by the median risk score in the E-MTAB-1980 cohort based on the nomogram; (j) time-dependent ROC curves measuring the predictive value of the risk score in the E-MTAB-1980 cohort based on the nomogram. (The statistical method was a log-rank test for a single factor, with only one test and the reported *P* value was the *P* value).

We then analyzed the model independence to determine whether it was independent of common clinical variables and could be used as an independent prognostic factor in ccRCC patients. The univariate Cox analysis results showed that the age (*P* < 0.001), tumor grade (*P* < 0.001), tumor stage (*P* < 0.001), T stage (*P* < 0.001), N stage (*P* = 0.049), M stage (*P* < 0.001), and risk score (*P* < 0.001) were significantly related to the prognosis of patients (Figure 8(a)). Multivariate Cox analysis showed that only the age (*P* = 0.010), grade (*P* = 0.024), stage (*P* < 0.001), and risk score (*P* < 0.001) were independent prognostic factors affecting patients' prognosis (Figure 8(b)).

Subsequently, to extend the clinical applicability of the MTG-based prognostic signature, based on the TCGA cohort, a nomogram containing a prognostic risk score and common clinical variables was constructed that could be conveniently used to calculate the expected survival of ccRCC patients (Figure 8(c)). The calibration curves at different time points showed that there is a good agreement between the predicted value and the true value (Figures 8(d)–8(f)). We further verified the accuracy and stability of the nomogram based on the TCGA cohort and E-MTAB-1980 cohort. Kaplan-Meier survival curve analysis indicated that risk stratification of ccRCC patients based on the nomogram could accurately distinguish patients with poor prognosis (*P* < 0.001 and *P* = 9.691*e* − 05, Figures 8(g) and 8(i)). In the TCGA cohort, the predicted AUC values for the nomogram at one, three, and five years were 0.862, 0.808, and 0.784, respectively (Figure 8(h)). And in the E-MTAB-1980 cohort, the predicted AUC values for the nomogram at one, three, and five years were 0.907, 0.894, and 0.884, respectively (Figure 8(j)). The above results indicated that the nomogram had good predictive ability and accuracy.

### 3.11. Assessment of Immune Cell Infiltration and Immunotherapy

**Figure 9:**
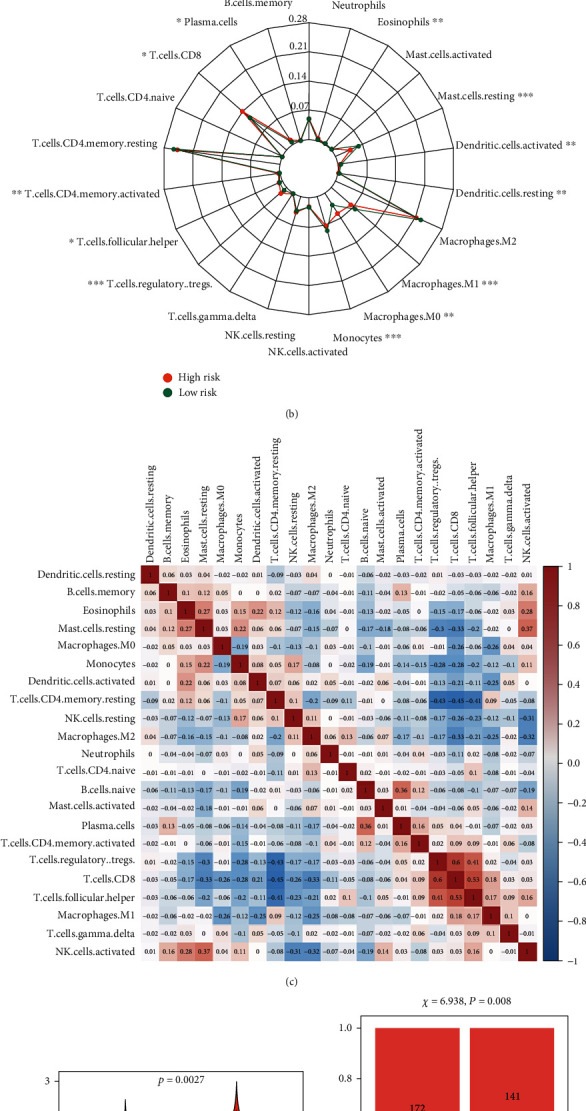
Assessment of immune cell infiltration and immunotherapy. (a) Stacked bar chart of the distribution of 22 immune cells in each ccRCC sample in the TCGA cohort; (b) radar plot of immune cell infiltration grouped by the median risk score in the TCGA cohort; (c) proportional correlation matrix of immune cell; (d) TIDE prediction score grouped by the median risk score in the TCGA cohort; (e) immunotherapeutic responses grouped by median risk score in the TCGA cohort. (The statistical method was *t*-test; the reported *P* value was the fdr value).

In addition to generating ATP through oxidative phosphorylation, mitochondria also play a crucial role in the integrity, proliferation, and growth of immune cells [28]. Mitochondria not only maintain the phenotype of immune cells but also are the necessary conditions for the establishment and function of their phenotypes [13]. Mitochondria can rapidly transform from catabolic organelles that produce ATP to synthetic organelles that produce ATP and macromolecule synthesis at the same time, which enables them to meet the appropriate metabolic requirements of different immune cells [29]. Moreover, targeting human mitochondrial metabolism has been shown to modulate immune responses in disease. For example, Singhal et al. [30] found that the antidiabetic drug metformin, which acts on mitochondrial complex I, is used as an immune modulator for tuberculosis. Therefore, in this study, we separately explored the effects on immune cell infiltration and immunotherapy. The degree of immune cell infiltration between high- and low-risk groups was assessed based on CIBERSORT and its gene set LM22. As shown in Figure 9(a), there were significant differences in the composition of the 22 immune cells in each sample. Specifically, there were significant differences in plasma cells, T cells CD8, T cell CD4 memory activated, T cell follicular helper, T cell regulatory Tregs, monocytes, macrophage M0, macrophages M1, dendritic cells resting, dendritic cells activated, mast cells resting, and eosinophils between the two groups (Figure 9(b)). The differences of immune cell infiltration among ccRCC patients stratified by different clinical characteristics are shown in Table S6. In addition, we also analyzed the difference in the infiltration of M2 macrophage marker CD163 between ccRCC and normal kidney tissue by immunofluorescence assay and the results showed that the infiltration level of CD163 in each tumor group was significantly higher than that in normal kidney tissue (Figure S8). And the clinical information of corresponding ccRCC patients was shown in Table S7. Correlation matrix results revealed that the T cell CD8 had the strongest positive correlation with T regulatory cells (Tregs) (Figure 9(c)). Subsequently, we predicted the likelihood of an immunotherapy response in ccRCC patients from the TCGA cohort based on a TIDE algorithm.  Figure 9(d) shows that the low-risk group had lower TIDE prediction score (*P* = 0.003).  Figure 9(e) shows that patients in the low-risk group had a higher response rate to immunotherapy (46.59% vs 35.34%, *P* = 0.008). These results provide further evidence that patients in the low-risk group have better prognosis and may have more potential for immunotherapy.

**Figure 10:**
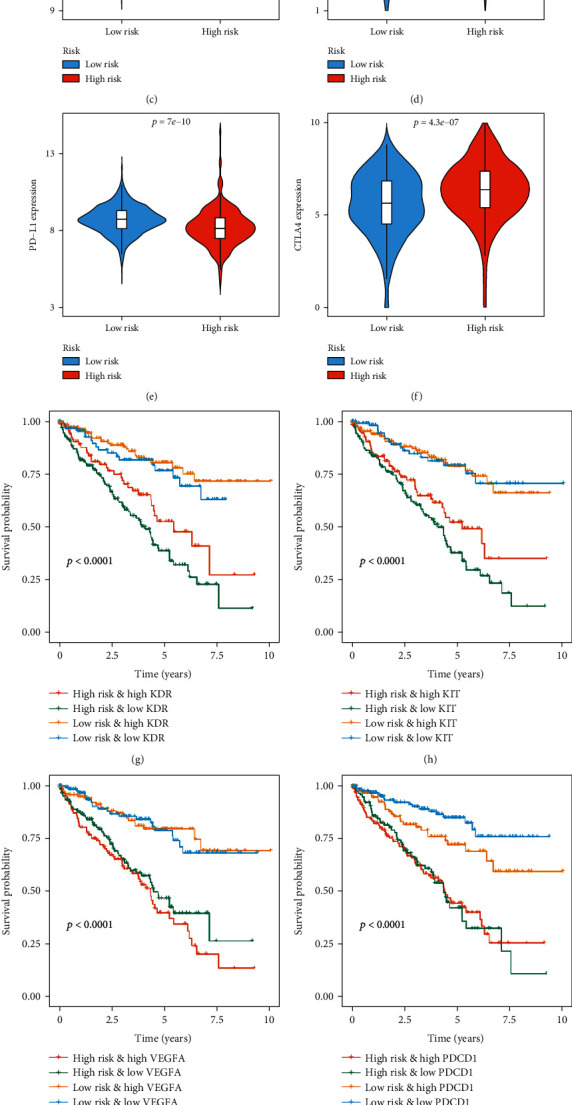
Expression of common immune checkpoint inhibitor genes and targeted genes under MTG-based prognostic signature stratification and their effect on clinical prognosis of patients with ccRCC. The expression levels of immune checkpoint genes and targeted genes were compared between high-risk and low-risk groups in TCGA cohort. (a) KDR; (b) KIT; (c) VEGFA; (d) PD-1; (e) PD-L1; (f) CTLA4. Kaplan-Meier survival curve analysis for overall survival among four patient groups grouped by the MTG-based prognostic signature and (g) KDR, (h) KIT, (i) VEGFA, (j) PD-1, (k) PD-L1, and (l) CTLA4. (The statistical method was a *t*-test with only one test and a log-rank test for a single factor, with only one test, and the reported *P* value was the *P* value).

Additionally, previous studies have shown that immune checkpoint inhibitor (ICI) genes can regulate immune cell infiltration in tumor tissues [31]. Thus, we also compared the expression levels of common ICI genes and angiogenesis-related genes (PD-1, PD-L1, CTLA4, KDR, KIT, and VEGFR) in ccRCC between different patient groups based on prognostic signature stratification. As shown in Figure 10, the expression levels of KDR (*P* < 0.001), KIT (*P* = 2.5*e* − 07), and PD-L1 (*P* = 7*e* − 10) in the low-risk group were significantly higher than those in the high-risk group. The expression levels of PD-1 (*P* < 0.001) and CTLA4 (*P* = 4.3*e* − 07) in the low-risk group were significantly lower than those in the high-risk group. However, there was no significant difference in VEGFA expression between the two groups. Next, we further investigated whether the MTG-based prognostic signature had any effect on clinical outcomes in patients with similar expression levels of these genes by comparing the survival distribution of the four patient groups stratified by the signature and the high/low gene expression. The results showed that patients with low risk and low KDR (or low risk and high KDR) had significantly better survival than those with high risk and low KDR (or high risk and high KDR) (*P* < 0.001, Figure 10(g)). Similar results were shown in KIT, VEGFA, PD-1, PD-L1, and CTLA4 (*P* < 0.001, Figures 10(h)–10(l)). We also observed that patients with low risk and low gene expression tended to have significantly better survival than patients in the other three groups. These results further suggest that the prognostic signature may be a potential marker of immunotherapeutic response in patients with ccRCC.

### 3.12. Relationship between the Prognostic MTG Expression Level and Drug Sensitivity of Various ccRCC Cell Lines

**Figure 11:**
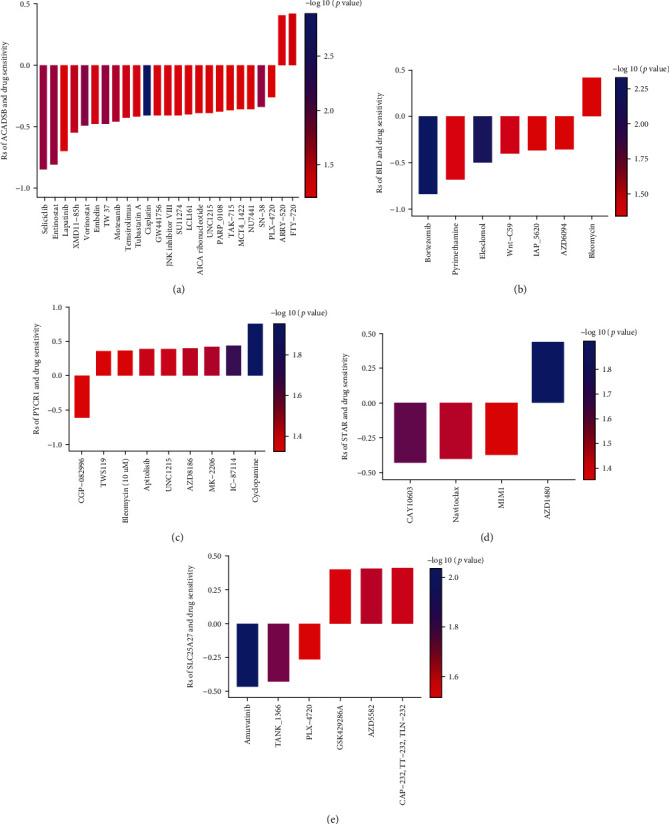
Relationship between the prognostic MTG expression level and drug sensitivity of ccRCC cell lines. The plot shows the correlation between the expression status of (a) ACADSB, (b) BID, (c) PYCR1, (d) STAR, and (e) SLC25A27 genes relative to the sensitivity of several ccRCC cell lines to various drugs. (The statistical method was Pearson correlation analysis. The reported *P* value was the fdr value).

Additionally, we analyzed the relationship between IC50 values of targeted drugs in various ccRCC cell lines and the expression levels of these prognostic MTGs using GDSC database. We believed that positive correlation indicates increased drug resistance of cell lines, while negative correlation indicates increased drug sensitivity of cell lines. The results showed that the high expression of ACADSB promoted the drug resistance of ccRCC cell lines to FTY 720 and ARRY-520 but enhanced the drug sensitivity of ccRCC cell lines to cisplatin, vorinostat, SN-38, and other drugs (Figure 11(a)). The high expression of BID promoted the drug resistance of ccRCC cell lines to bleomycin but enhanced the drug sensitivity of ccRCC cell lines to bortezomib, elesclomol, Wnt-C59, and other drugs (Figure 11(b)). The high expression of PYCR1 promoted the drug resistance of ccRCC cell lines to cyclopamine, IC-87114, MK-2206, and other drugs but enhanced the drug sensitivity of ccRCC cell lines to CGP-082996 (Figure 11(c)). The high expression of STAR promoted the drug resistance of ccRCC cell lines to AZD1480 but enhanced the drug sensitivity of ccRCC cell lines to CAY10603, navitoclax, MIM1, and other drugs (Figure 11(d)). The high expression of SLC25A27 promoted the drug resistance of ccRCC cell lines to GSK429286A, AZD5582, CAP-232, TT-232, and TLN-232 but enhanced the drug sensitivity of ccRCC cell lines to amuvatinib, TANK_1366, and PLX-4720 (Figure 11(e)).

### 3.13. Prognostic Potential of the MTG-Based Signature in chRCC

ChRCC is another subtype of RCC, accounting for about 5% [32]. The aberration and overexpression of mitochondrial DNA are considered to be the main characteristics of chRCC tissues [33–35]. Davis et al. [33] found an increase in the importance of a distinct mitochondrial respiratory program in this disease, and an increase in oxidative phosphorylation is maintained in complex I-altered tumors, indicating a metabolic shift supporting for the growth of this tumor. Considering that mitochondrial dysregulation plays an important role in chRCC, we further explored the prognostic value of the MTG-based signature in chRCC. The expression matrix of 65 patients with chRCC containing OS, DFS, DSS, PFS, and other clinical prognostic information was obtained from the cBioPortal database. We used the same formula to calculate the risk score for each chRCC patient. Similarly, patients with chRCC were divided into low-risk and high-risk groups based on the median risk score. Kaplan-Meier survival analysis showed that high-risk chRCC patients had significantly lower OS (*P* = 0.017, Figure S9A), DFS (*P* = 0.029, Figure S9C), DSS (*P* = 0.007, Figure S9E), and PFS (*P* = 0.013, Figure S9G) than low-risk chRCC patients. The predicted AUC values also showed that the prognostic signature has good predictive performance (Figure S9B, D, F, H). These results showed that the MTG-based signature also had an important value in predicting the prognosis of patients with chRCC.

### 3.14. Verification of the Expression Levels of the Target Genes in ccRCC and Normal Renal Tissues by RT-qPCR

**Figure 12:**
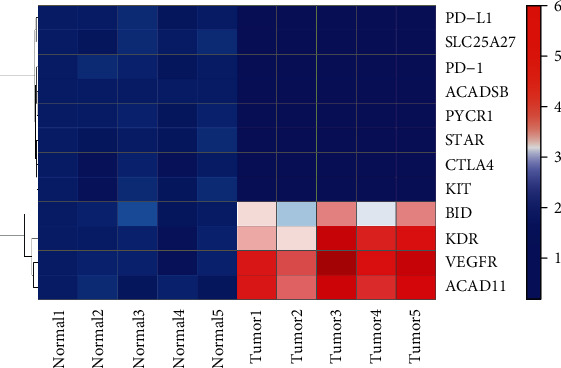
The expression heatmap of the target genes in in ccRCC and adjacent nontumor renal tissues.

The actual expression levels of common ICI genes (KDR, KIT, and VEGFR), angiogenesis-related genes (PD-1, PD-L1, and CTLA4), and prognostic-related MTGs (ACAD11, ACADSB, BID, PYCR1, SLC25A27, and STAR) in ccRCC and normal renal tissues were detected by RT-qPCR to further evaluate the reliability of the prognostic signature. The analysis results are shown in Figure 12. The mRNA expression of KIT, PD-1, PD-L1, CTLA4, ACADSB, PYCR1, SLC25A27, and STAR in ccRCC tissues was significantly lower than that in adjacent nontumor renal tissues. However, the mRNA expression of KDR, VEGFR ACAD11, and BID in ccRCC tissues was significantly higher than that in adjacent nontumor renal tissues.

## 4. Discussion

Mitochondria are primarily involved in bioenergy metabolism and cellular homeostasis, including the production of ATP through oxidative phosphorylation, the decomposition of fatty acids through *β* oxidation, the production of reactive oxygen species, and the initiation and execution of apoptosis [36, 37]. There are also multiple mtDNA copies in mitochondria, which mainly encode rRNAs, tRNAs, and proteins necessary for electron transport and oxidative phosphorylation, as well as the genetic repair mechanism of mitochondria [38, 39]. Mitochondrial dysfunction is thought to be a hallmark of many diseases. Notably, cellular metabolic reprogramming and imbalance of the redox system have also been recognized as major markers of tumor transformation [40]. Mitochondrial dysfunction caused by a variety of reasons including the mtDNA mutations, mitochondrial respiratory chain enzyme dysfunction, oxidative stress, and cancer or tumor suppressor signals can change cell metabolic pathways, can destroy the intracellular redox homeostasis, and leads to cell apoptosis and treatment resistance, which damage the cell steady state, promoting the development of genetic instability and cancer [15, 40]. At present, most research on mitochondria has focused on the function of individual genes. Few studies have used expression profile data to systematically explore the molecular characteristics and prognostic potential of MTGs.

With the development of bioinformatics technology, many methods can be used to screen and identify key genes, including the classic differential analysis and the recently popular WGCNA analysis. Herein, as we have been using the traditional method of difference analysis, guided by TCGA cohort transcriptome analysis, we identified 114 differentially expressed MTGs associated with oxidative stress. Functional enrichment analysis of these MTGs was then performed to understand their biological functions and molecular mechanisms. Next, the Cox regression analysis was used to identify 11 prognostic MTGs and build a signature. We further explored the correlation between the prognostic MTGs and signature with common clinical variables, upstream regulatory mechanisms, immune cell infiltration and immunotherapy, and drug sensitivity of various ccRCC cell lines.

After univariate Cox, LASSO, and multivariate Cox regression analyses, 11 genes were identified which were most associated with prognosis, including ACAD11, ACADSB, BID, PYCR1, SLC25A27, and STAR. As a member of the acyl-CoA dehydrogenase family, ACAD11 is highly expressed in a variety of human organs, including the kidney, and plays an important role in energy homeostasis in pathophysiological processes [41]. Jiang et al. [42] also found that ACAD11 acts as an important metabolic target of p53 during its prosurvival function. As a member of the acyl-CoA dehydrogenase family, the main function of ACADSB is to dehydrogenate acyl-CoA derivatives. It has been reported that ACADSB is low expressed in poorly differentiated hepatocellular carcinoma cells [43]. Zhang et al. [44] also showed that ACADSB was low expressed in ccRCC tissues and could serve as a potential target for diagnosis and treatment of ccRCC. BID is one of the only proteins in BH3 that control BAK and BAX and is believed to play a key role in the apoptotic signaling pathway [45]. PYCR1 is a mitochondrial intimal protein that is the rate-limiting step in proline synthesis. PYCR1 has been reported to be upregulated in many human cancers, including prostate cancer, thereby promoting cancer progression [46]. Zhuang et al. [47] found that the downregulation of PYCR1 inhibited the proliferation of hepatocellular carcinoma cells and promoted apoptosis by inhibiting the JNK/IRS1 pathway. SLC25A27 encodes uncoupling protein-4 (UCP4), a member of the larger mitochondrial anion carrier protein family. Chu et al. [48] found that overexpression of UCP4 in SH-SY5Y neuroblastoma cells reduced oxidative stress but also unexpectedly increased cellular ATP levels. Ho et al. [49] showed that UCP4 promotes cancer cell growth in neuroblastoma cells by interacting with mitochondrial complex II to increase ATP supply. STAR regulates steroid hormone biosynthesis by promoting cholesterol conversion to pregnenolone [50]. Studies have shown that malfunctions in steroid production mechanisms involving androgen or estrogen biosynthesis are associated with the pathogenesis of many malignancies [51]. Moreover, in our study, GSEA analysis and Pearson correlation analysis further revealed that these 6 MTGs may affect redox homeostasis, energy production, immune infiltration, RNA modification, and metastasis mechanism of ccRCC. These results suggest that these MTGs play an important role in many tumors and may be involved in the tumorigenesis and progression of ccRCC. However, further experiments are needed to explore the specific functions and molecular mechanisms of these MTGs.

Subsequently, we developed a prognostic signature based on these 11 MTGs. The Kaplan-Meier method showed that patients in the high-risk group had significantly shorter OS. The ROC curve indicated that the signature had high accuracy in predicting 1-, 3- and 5-year survival rates. We found that the signature could accurately distinguish patients with poor prognosis under different clinical variable stratification. The prognostic risk score was also associated with disease progression in ccRCC tumors.

Additionally, we also revealed the upstream regulatory network of these prognostic genes and identified 32 TFs involved in the regulation of these genes, which are worthy of further study. GO and KEGG analysis indicated that these differentially expressed MTGs were mainly concentrated in metabolic processes of biomolecules such as lipids, proteins, nucleic acids, apoptosis pathways, and redox processes. Indeed, mitochondria as an important organelle produce energy and participate in a variety of biological processes; its dysfunction can cause the TCA cycle enzyme destruction, electronic respiratory chain leakage, and the subsequent oxidative stress, which change the cell metabolism and signal transduction pathways, leading to the resistance to apoptosis and treatment, significantly promoting the development of a variety of human cancers [40]. At the same time, mitochondria are also the core of immunity, which determines the fate of immunity. Mitochondria connect various metabolic pathways to every subpopulation of immune cells, from T cells to macrophages [12]. Our results also found significant differences in immune cell infiltration and immunotherapy response rates between the high-risk and low-risk groups. Besides, the relationship between the expression levels of these MTGs and the drug sensitivity of various ccRCC cell lines was also analyzed, which may help guide clinical treatment.

In recent years, with the progress of biomolecular science, more and more new molecular biomarkers have been developed and provided new insights for the biology of ccRCC. This has led to the development of cancer-related biomarkers as well as new targeted therapies including proliferation markers such as Ki-67, p53, and PTEN; the hypoxia-inducible factor pathway; carbonic anhydrase IX; and vascular endothelial growth factor (VEGF) [52]. Various prognostic signatures have been developed from the perspectives of somatic mutations, gene methylation differences, gene expression differences, posttranscriptional modification differences, and immune pathways of ccRCC [53–55]. Chen et al. [7] established a three-gene signature to predict prognosis of ccRCC. Chen et al. [8] developed a seven-gene signature to predict OS of ccRCC. Few signatures have been used clinically. Hua et al. systematically studied the immune-related phenotypes of ccRCC and their relationship with prognosis and developed a signature of five immune-related genes to predict the prognosis of ccRCC [56]. Marquardt et al. [17] also recently proposed a unique histologically independent subgroup, the mixed subgroup, characterized by enhanced mitochondria and weakened angiogenesis-related genetic signatures. In our study, we systematically analyzed the biological functions and molecular mechanisms of MTGs in ccRCC and developed a new prognostic signature based on 6 MTGs, which was different from the existing signatures and further advanced the research of Marquardt et al.

Overall, this study provides a new understanding of the tumorigenesis and progression of ccRCC from the perspective of mitochondria. These 6 MTG-based prognostic signature could accurately distinguish ccRCC patients with poor prognosis. Moreover, these prognostic MTGs have important biological functions and clinical value. Nevertheless, our study still has some limitations. On the one hand, our model is based on retrospective analysis and needs to be validated in prospective studies. On the other hand, this study is mainly based on the analysis of bioinformatics technology, but the functional mechanism and interaction of genes are complex and more experimental data are needed for verification and evaluation. Moreover, although the low-risk group had a better immunotherapy response, this result is based on bioinformatics predictions and requires further validation in prospective cohorts.

## 5. Conclusions

We have systematically investigated the molecular characteristics of MTGs associated with oxidative stress and their prognostic potential. We have also revealed the complex biological functions and regulatory networks of these MTGs, which will contribute to the further understanding of the molecular mechanisms involved in ccRCC tumorigenesis and progression. Besides, we developed a new prognostic signature that can accurately distinguish patients with poor prognosis. This will enrich the treatment strategy for patients with ccRCC and provide certain guidance for clinical work from the perspective of targeting mitochondria.

## Figures and Tables

**Table 1:** The relationship between prognostic-related mitochondrial genes and clinicopathologic parameters.

Gene		Gender (male/female)	Grade (G1–2/G3–4)	Stage (I–II/III–IV)	T stage (T1–T2/T3–T4)	N stage (N0/NI–X)	M stage (M0/MI–X)
N		353/186	249/282	331/205	349/190	241/298	428/109
ACAD11	*t* value	3.637	2.16	2.979	2.359	0.149	NA^∗^
	*P* value	<0.001	0.031	0.003	0.019	0.881	<0.001
ACADSB	*t* value	1.475	NA^∗^	NA^∗^	6.841	0.135	4.957
	*P* value	0.141	<0.001	<0.001	<0.001	0.893	<0.001
BID	*t* value	2.926	NA^∗^	6.911	6.304	0.558	3.497
	*P* value	0.004	<0.001	<0.001	<0.001	0.577	0.001
PYCR1	*t* value	3.342	NA^∗^	NA^∗^	5.099	0.032	3.09
	*P* value	0.001	<0.001	<0.001	<0.001	0.974	0.002
SLC25A27	*t* value	1.392	3.023	NA^∗^	NA^∗^	0.749	0.193
	*P* value	0.165	0.003	0.009	0.037	0.455	0.847
STAR	*t* value	0.655	NA^∗^	4.251	4.485	0.491	3.346
	*P* value	0.513	0.007	<0.001	<0.001	0.624	0.001

NA: not available; ^∗^nonparametric Mann–Whitney rank sum test. (The statistical method was *t*-test or nonparametric Mann–Whitney rank sum test with only one test. The reported *P* value was the fdr value).

## Data Availability

The data and materials can be obtained by contacting the corresponding author.

## Abbreviations

RCC: Renal cell carcinoma
CcRCC: Clear cell renal cell carcinoma
ChRCC: Chromophobe renal cell carcinoma
TMM: Trimmed mean of *M* values
mtDNA: Mitochondrial DNA
mtROS: Mitochondrial reactive oxygen species
ROS: Reactive oxygen species
MTGs: Mitochondrial genes
TCGA: The Cancer Genome Atlas
GEO: Gene Expression Omnibus
RMA: Robust multichip average
FC: Fold change
FDR: False discovery rate
OS: Overall survival
DFS: Disease-free survival
DSS: Disease-specific survival
PFS: Progression-free survival
WGCNA: Weighted correlation network analysis
GSEA: Gene set enrichment analysis
m6A: N6-Methyladenosine
LASSO: Least absolute shrinkage and selection operator
ROC: Receiver operating characteristic
TFs: Transcription factors
GO: Gene Ontology
KEGG: Kyoto Encyclopedia of Genes and Genomes
AUC: Area under the receiver operating characteristic curve
CIBERSORT: Cell type identification by estimating relative subsets of RNA transcripts
TIDE: Tumor immune dysfunction and exclusion
MQC: Mitochondrial quality control
TFEB: Transcription factor EB
FOXO: Forkhead box O
Nrf2: Nuclear factor, erythroid 2-like 2
SOD2: Superoxide dismutase 2
ICI: Immune checkpoint inhibitor
UCP4: Uncoupling protein-4.

## Supplementary Materials

Supplementary Materials Supplemental Table S1: the sequences of primer RNA. Supplemental Table S2: univariate Cox regression analysis of differentially expressed MTGs. Supplemental Table S3: clinical characteristics of high- and low-risk patients. Supplemental Table S4: regulatory relationships between these TFs and prognostic related MTGs. Supplemental Table S5: the binding sites of transcription factors in the promoter region of prognostic MTGs were predicted by JASPAR database. Supplemental Table S6: difference of immune cell infiltration in patients with ccRCC under different clinical characteristics stratification. Supplemental Table S7: the clinical information of corresponding ccRCC patients. Supplemental Figure S1: relationship between gene modules and clinical variables based on WGCNA analysis. Supplemental Figure S2: ROC curve analysis of prognostic MTGs and signature. Supplemental Figure S3: GSEA of prognostic MTGs. Supplemental Figure S4: correlation of prognostic MTGs with immune infiltration in ccRCC. Supplemental Figure S5: correlation of prognostic MTGs with RNA modification in ccRCC. Supplemental Figure S6: RT-qPCR results were obtained after transfection of PBX1-overexpressed plasmid and interference plasmid. Supplemental Figure S7: functional enrichment analysis of TFs. Supplemental Figure S8: immunofluorescence analysis of M2 macrophage marker CD163 in ccRCC and normal renal tissue. Supplemental Figure S9: prognostic potential of the MTG-based signature in chRCC.

## References

[1] Buti S., Bersanelli M., Sikokis A. (2013). Chemotherapy in metastatic renal cell carcinoma today? A systematic review. *Anti-Cancer Drugs*.

[2] Hakimi A. A., Pham C. G., Hsieh J. J. (2013). A clear picture of renal cell carcinoma. *Nature Genetics*.

[3] Karakiewicz P. I., Briganti A., Chun F. K. (2007). Multi-institutional validation of a new renal cancer-specific survival nomogram. *Journal of Clinical Oncology*.

[4] Nerich V., Hugues M., Nai T. (2014). Clinical impact of targeted therapies in patients with metastatic clear-cell renal cell carcinoma. *Oncotargets and Therapy*.

[5] Muselaers C. H., Boerman O. C., Oosterwijk E., Langenhuijsen J. F., Oyen W. J., Mulders P. F. (2013). Indium-111-labeled girentuximab immunoSPECT as a diagnostic tool in clear cell renal cell carcinoma. *European Urology*.

[6] Siegel R. L., Miller K. D., Jemal A. (2017). Cancer statistics, 2017. *CA: a Cancer Journal for Clinicians*.

[7] Chen L., Luo Y., Wang G. (2019). Prognostic value of a gene signature in clear cell renal cell carcinoma. *Journal of Cellular Physiology*.

[8] Chen L., Xiang Z., Chen X., Zhu X., Peng X. (2020). A seven-gene signature model predicts overall survival in kidney renal clear cell carcinoma. *Hereditas*.

[9] Argentiero A., Solimando A. G., Krebs M. (2020). Anti-angiogenesis and immunotherapy: novel paradigms to envision tailored approaches in renal cell-carcinoma. *Journal of Clinical Medicine*.

[10] Newmeyer D. D., Ferguson-Miller S. (2003). Mitochondria: releasing power for life and unleashing the machineries of death. *Cell*.

[11] Lu H., Li G., Liu L., Feng L., Wang X., Jin H. (2013). Regulation and function of mitophagy in development and cancer. *Autophagy*.

[12] Mehta M. M., Weinberg S. E., Chandel N. S. (2017). Mitochondrial control of immunity: beyond ATP. *Nature Reviews. Immunology*.

[13] Rambold A. S., Pearce E. L. (2018). Mitochondrial dynamics at the interface of immune cell metabolism and function. *Trends in Immunology*.

[14] Figueira T. R., Barros M. H., Camargo A. A. (2013). Mitochondria as a source of reactive oxygen and nitrogen species: from molecular mechanisms to human health. *Antioxidants & Redox Signaling*.

[15] Zhou Y., Hileman E. O., Plunkett W., Keating M. J., Huang P. (2003). Free radical stress in chronic lymphocytic leukemia cells and its role in cellular sensitivity to ROS-generating anticancer agents. *Blood*.

[16] Gardner P. R., Nguyen D. D., White C. W. (1994). Aconitase is a sensitive and critical target of oxygen poisoning in cultured mammalian cells and in rat lungs. *Proceedings of the National Academy of Sciences*.

[17] Marquardt A., Solimando A. G., Kerscher A. (2021). Subgroup-Independent Mapping of Renal Cell Carcinoma-Machine Learning Reveals Prognostic Mitochondrial Gene Signature Beyond Histopathologic Boundaries. *Frontiers in oncology*.

[18] Wagner G. P., Kin K., Lynch V. J. (2012). Measurement of mRNA abundance using RNA-seq data: RPKM measure is inconsistent among samples. *Theory in Biosciences*.

[19] Rath S., Sharma R., Gupta R. (2021). MitoCarta3.0: an updated mitochondrial proteome now with sub-organelle localization and pathway annotations. *Nucleic Acids Research*.

[20] Liu T., Ortiz J. A., Taing L. (2011). Cistrome: an integrative platform for transcriptional regulation studies. *Genome Biology*.

[21] Newman A. M., Liu C. L., Green M. R. (2015). Robust enumeration of cell subsets from tissue expression profiles. *Nature Methods*.

[22] Jiang P., Gu S., Pan D. (2018). Signatures of T cell dysfunction and exclusion predict cancer immunotherapy response. *Nature Medicine*.

[23] Barbieri I., Kouzarides T. (2020). Role of RNA modifications in cancer. *Nature Reviews. Cancer*.

[24] Liesa M., Shirihai O. S. (2013). Mitochondrial dynamics in the regulation of nutrient utilization and energy expenditure. *Cell Metabolism*.

[25] Wang S., Chen Y., Li X. (2020). Emerging role of transcription factor EB in mitochondrial quality control. *Biomedicine & Pharmacotherapy*.

[26] Kim S., Koh H. (2017). Role of FOXO transcription factors in crosstalk between mitochondria and the nucleus. *Journal of Bioenergetics and Biomembranes*.

[27] Ryoo I. G., Kwak M. K. (2018). Regulatory crosstalk between the oxidative stress-related transcription factor Nfe2l2/Nrf2 and mitochondria. *Toxicology and Applied Pharmacology*.

[28] Alissafi T., Kalafati L., Lazari M. (2020). Mitochondrial oxidative damage underlies regulatory T cell defects in autoimmunity. *Cell Metabolism*.

[29] Weinberg S. E., Sena L. A., Chandel N. S. (2015). Mitochondria in the regulation of innate and adaptive immunity. *Immunity*.

[30] Singhal A., Jie L., Kumar P. (2014). Metformin as adjunct antituberculosis therapy. *Science Translational Medicine*.

[31] Sun J., Zhang Z., Bao S. (2020). Identification of tumor immune infiltration-associated lncRNAs for improving prognosis and immunotherapy response of patients with non-small cell lung cancer. *Journal for Immunotherapy of Cancer*.

[32] Störkel S., Eble J. N., Adlakha K. (1997). Classification of renal cell carcinoma. *Cancer*.

[33] Davis C. F., Ricketts C. J., Wang M. (2014). The somatic genomic landscape of chromophobe renal cell carcinoma. *Cancer Cell*.

[34] Kovacs A., Storkel S., Thoenes W., Kovacs G. J. (1992). Mitochondrial and chromosomal DNA alterations in human chromophobe renal cell carcinomas. *The Journal of pathology*.

[35] Nagy A., Wilhelm M., Sükösd F., Ljungberg B., Kovacs G. (2002). Somatic mitochondrial DNA mutations in human chromophobe renal cell carcinomas. *Genes Chromosomes Cancer*.

[36] Wallace D. C., Fan W., Procaccio V. (2010). Mitochondrial energetics and therapeutics. *Annual Review of Pathology*.

[37] Galluzzi L., Kepp O., Kroemer G. (2012). Mitochondria: master regulators of danger signalling.

[38] Wallace D. C. (1999). Mitochondrial diseases in man and mouse. *Science*.

[39] Bonawitz N. D., Clayton D. A., Shadel G. S. (2006). Initiation and beyond: multiple functions of the human mitochondrial transcription machinery.

[40] Luo Y., Ma J., Lu W. (2020). The Significance of Mitochondrial Dysfunction in Cancer. *International Journal of Molecular Sciences*.

[41] He M., Pei Z., Mohsen A. W. (2011). Identification and characterization of new long chain acyl-CoA dehydrogenases. *Molecular Genetics and Metabolism*.

[42] Jiang D., LaGory E. L., Kenzelmann Brož D. (2015). Analysis of p53 transactivation domain mutants reveals _Acad11_ as a metabolic target important for p53 pro-survival function. *Cell Reports*.

[43] Nwosu Z. C., Battello N., Rothley M. (2018). Liver cancer cell lines distinctly mimic the metabolic gene expression pattern of the corresponding human tumours. *Journal of Experimental & Clinical Cancer Research*.

[44] Zhang B., Wu Q., Wang Z. (2019). The promising novel biomarkers and candidate small molecule drugs in kidney renal clear cell carcinoma: evidence from bioinformatics analysis of high- throughput data. *Molecular Genetics & Genomic Medicine*.

[45] König H. G., Rehm M., Gudorf D. (2007). Full length bid is sufficient to induce apoptosis of cultured rat hippocampal neurons. *BMC Cell Biology*.

[46] Zeng T., Zhu L., Liao M. (2017). Knockdown of PYCR1 inhibits cell proliferation and colony formation via cell cycle arrest and apoptosis in prostate cancer. *Medical Oncology*.

[47] Zhuang J., Song Y., Ye Y. (2019). PYCR1 interference inhibits cell growth and survival via c-Jun N-terminal kinase/insulin receptor substrate 1 (JNK/IRS1) pathway in hepatocellular cancer. *Journal of Translational Medicine*.

[48] Chu A. C., Ho P. W., Kwok K. H. (2009). Mitochondrial UCP4 attenuates MPP+- and dopamine-induced oxidative stress, mitochondrial depolarization, and ATP deficiency in neurons and is interlinked with UCP2 expression. *Free Radical Biology and Medicine*.

[49] Ho P. W., Ho J. W., Tse H. M. (2012). Uncoupling protein-4 (UCP4) increases ATP supply by interacting with mitochondrial complex II in neuroblastoma cells. *PLoS One*.

[50] Manna P. R., Stetson C. L., Slominski A. T., Pruitt K. (2016). Role of the steroidogenic acute regulatory protein in health and disease. *Endocrine*.

[51] Folkerd E., Dowsett M. (2013). Sex hormones and breast cancer risk and prognosis. *Breast*.

[52] Sun M., Shariat S. F., Cheng C. (2011). Prognostic factors and predictive models in renal cell carcinoma: a contemporary review. *European Urology*.

[53] de Martino M., Haitel A., Schatzl G., Klatte T. (2013). The protease activated receptor 1 gene variation IVSn –14 A>T is associated with distant metastasis and cancer specific survival in renal cell carcinoma. *The Journal of Urology*.

[54] Stewart G. D., O'Mahony F. C., Laird A. (2014). Carbonic anhydrase 9 expression increases with vascular endothelial growth factor-targeted therapy and is predictive of outcome in metastatic clear cell renal cancer. *European Urology*.

[55] Sim S. H., Messenger M. P., Gregory W. M. (2012). Prognostic utility of pre-operative circulating osteopontin, carbonic anhydrase IX and CRP in renal cell carcinoma. *British Journal of Cancer*.

[56] Hua X., Chen J., Su Y., Liang C. (2020). Identification of an immune-related risk signature for predicting prognosis in clear cell renal cell carcinoma. *Aging*.

