# Supplementary material for: A Mitochondrial Dysfunction and Oxidative Stress Pathway-Based Prognostic Signature for Clear Cell Renal Cell Carcinoma
Source: Oxid Med Cell Longev. 2021 Nov 24;2021:9939331. doi: 10.1155/2021/9939331 (PMC8635875; doi:10.1155/2021/9939331)
Supplement: Supplementary Materials — Supplemental Table S1: the sequences of primer RNA. Supplemental Table S2: univariate Cox regression analysis of differentially expressed MTGs. Supplemental Table S3: clinical characteristics of high- and low-risk patients. Supplemental Table S4: regulatory relationships between these TFs and prognostic related MTGs. Supplemental Table S5: the binding sites of transcription factors in the promoter region of prognostic MTGs were predicted by JASPAR database. Supplemental Table S6: difference of immune cell infiltration in patients with ccRCC under different clinical characteristics stratification. Supplemental Table S7: the clinical information of corresponding ccRCC patients. Supplemental Figure S1: relationship between gene modules and clinical variables based on WGCNA analysis. Supplemental Figure S2: ROC curve analysis of prognostic MTGs and signature. Supplemental Figure S3: GSEA of prognostic MTGs. Supplemental Figure S4: correlation of prognostic MTGs with immune infiltration in ccRCC. Supplemental Figure S5: correlation of prognostic MTGs with RNA modification in ccRCC. Supplemental Figure S6: RT-qPCR results were obtained after transfection of PBX1-overexpressed plasmid and interference plasmid. Supplemental Figure S7: functional enrichment analysis of TFs. Supplemental Figure S8: immunofluorescence analysis of M2 macrophage marker CD163 in ccRCC and normal renal tissue. Supplemental Figure S9: prognostic potential of the MTG-based signature in chRCC. [file 9939331.f1.docx]

**A mitochondrial dysfunction and oxidative stress pathway-based prognostic signature for clear cell renal cell carcinoma**

Table S1. The sequences of primer RNA.

| Name | Primer | Sequence |
| --- | --- | --- |
| Human GAPDH | Forward | 5‘- GAGAAGGCTGGGGCTCATTT-3’ |
|  | Reverse | 5‘- AGTGATGGCATGGACTGTGG-3’ |
| Human PD-1 | Forward | 5‘- CTTGCCTTCAAGCCTTCTGC-3’ |
|  | Reverse | 5‘- ACCACACTGTCGTCGAATGG-3’ |
| Human PD-L1 | Forward | 5‘- AGACCACCACCACCAATTCC-3’ |
|  | Reverse | 5‘- GCCAGAGGTAGTTCTGGGATG-3’ |
| Human CTLA4 | Forward | 5‘- CAAGTGCCTTCTGTGTGTGC-3’ |
|  | Reverse | 5‘- GAGCGGTGTTCAGGTCTTCA-3’ |
| Human KDR | Forward | 5‘- CGGTCAACAAAGTCGGGAGA-3’ |
|  | Reverse | 5‘- CAGTGCACCACAAAGACACG-3’ |
| Human KIT | Forward | 5‘- TGCTCTGCTTCTGTACTGCC-3’ |
|  | Reverse | 5‘- GCCTTACATTCAACCGTGCC-3’ |
| Human VEGFR | Forward | 5‘- AGACCGGCTGAAGCTAGGTA -3’ |
|  | Reverse | 5‘- GAGCTCGATGCTCACTGTGT -3’ |
| Human ACAD11 | Forward | 5‘- GTCCCTGGAGGCCTACCTAA-3’ |
|  | Reverse | 5‘- GGAAGAAGTGAACCTGGTGGT-3’ |
| Human ACADSB | Forward | 5‘- AGAGTTCAGTTGATGGGTATTGA-3’ |
|  | Reverse | 5‘- CTCTATCACGAGCACAGTGGA-3’ |
| Human BID | Forward | 5‘- AAGGAGGAAGCGGGTAGTCG -3’ |
|  | Reverse | 5‘- GGAACCGTTGTTGACCTCAC -3’ |
| Human PYCR1 | Forward | 5‘- CGCAGCGCTGAGGGAG-3’ |
|  | Reverse | 5‘- TCTTGTGGGCAGCCAAGAC-3’ |
| Human SLC25A27 | Forward | 5‘- CTTGGCTGAGAATGTGATCTGG-3’ |
|  | Reverse | 5‘- GCAACTCAAAGGCCAAACCT-3’ |
| Human STAR | Forward | 5‘- CAGACTTCGGGAACATGCCT-3’ |
|  | Reverse | 5‘- GGGACAGGACCTGGTTGATG-3’ |

Table S2. Univariate Cox regression analysis of differentially expressed MTGs.

| Gene | Hazard ratio | P value | Gene | Hazard ratio | P value |
| --- | --- | --- | --- | --- | --- |
| ABAT | 0.765 | <0.001 | FKBP10 | 1.430 | <0.001 |
| ABCB6 | 1.204 | 0.045 | GATM | 0.836 | <0.001 |
| ABCD1 | 1.810 | <0.001 | GLDC | 0.962 | 0.316 |
| ACAA1 | 0.797 | 0.131 | GUK1 | 1.389 | 0.016 |
| ACAD11 | 0.789 | <0.001 | HMGCS2 | 0.890 | <0.001 |
| ACADSB | 0.530 | <0.001 | HTATIP2 | 1.617 | <0.001 |
| ACLY | 0.812 | 0.005 | IFI27 | 1.070 | 0.348 |
| ACOT11 | 0.856 | 0.072 | LDHB | 1.004 | 0.979 |
| AGMAT | 0.897 | 0.004 | MRPL53 | 1.686 | <0.001 |
| AGXT | 0.915 | 0.031 | MRPS6 | 1.322 | 0.001 |
| AKR1B10 | 1.093 | <0.001 | MTHFD2 | 1.561 | <0.001 |
| ALDH1L2 | 1.263 | <0.001 | OGDHL | 0.817 | <0.001 |
| ALDH4A1 | 0.924 | 0.200 | OTC | 0.930 | 0.214 |
| ARG2 | 1.063 | 0.133 | PCCB | 1.055 | 0.664 |
| ATAD3B | 1.504 | <0.001 | PCK2 | 0.799 | 0.031 |
| BAX | 1.437 | 0.012 | PDK1 | 0.881 | 0.159 |
| BBC3 | 1.318 | 0.011 | POLG2 | 1.522 | <0.001 |
| BCL2A1 | 1.259 | <0.001 | POLQ | 1.373 | <0.001 |
| BCL2L10 | 0.982 | 0.616 | PRDX4 | 1.046 | 0.745 |
| BDH1 | 1.061 | 0.257 | PRODH2 | 0.962 | 0.174 |
| BID | 2.731 | <0.001 | PYCR1 | 1.409 | <0.001 |
| BNIP3 | 0.750 | <0.001 | RDH13 | 1.027 | 0.669 |
| CBR3 | 1.285 | 0.001 | SFXN3 | 1.315 | 0.036 |
| COX4I2 | 0.935 | 0.194 | SHMT2 | 1.192 | 0.132 |
| CPT1B | 1.193 | <0.001 | SLC25A27 | 1.127 | 0.021 |
| CYP27B1 | 1.196 | 0.001 | SOD2 | 1.214 | 0.010 |
| DCXR | 1.271 | 0.006 | STAR | 1.331 | <0.001 |
| EFHD1 | 0.810 | 0.002 | SUCLG1 | 0.700 | 0.119 |
| FABP1 | 0.927 | 0.016 | TAZ | 1.980 | <0.001 |
| FDXR | 0.998 | 0.986 | TRMT1 | 1.917 | <0.001 |
| FECH | 0.506 | <0.001 | UCP3 | 1.326 | <0.001 |

Table S3. Clinical characteristics of high- and low-risk patients.

|  |  | Low risk | High risk |
| --- | --- | --- | --- |
| Age | ≤65 | 178 | 175 |
|  | >65 | 92 | 94 |
| Gender | Male | 159 | 194 |
|  | Female | 111 | 75 |
| Grade | Grade1-2 | 149 | 100 |
|  | Grade 3-4 | 114 | 168 |
| Stage | Stage Ⅰ-Ⅱ | 194 | 137 |
|  | Stage Ⅲ-Ⅳ | 75 | 130 |
| T stage | T 1-2 | 201 | 148 |
|  | T 3-4 | 69 | 121 |
| N stage | N0 | 125 | 116 |
|  | N1 | 4 | 12 |
|  | NX | 141 | 141 |
| M stage | M0 | 233 | 195 |
|  | M1 | 22 | 56 |
|  | MX | 15 | 16 |
| Status | Alive | 225 | 148 |
|  | Dead | 45 | 121 |

Table S4. Regulatory relationships between these TFs and prognostic related MTGs.

| Transcription factors | Mitochondrial genes | Coefficient | P value | Regulation |
| --- | --- | --- | --- | --- |
| ETS1 | ACAD11 | 0.359 | 8.00E-18 | Positive |
| CEBPB | ACAD11 | -0.366 | 1.64E-18 | Negative |
| PML | ACADSB | -0.480 | 2.39E-32 | Negative |
| BATF | ACADSB | -0.419 | 2.20E-24 | Negative |
| PBX1 | ACADSB | 0.424 | 5.86E-25 | Positive |
| MEF2B | ACADSB | -0.305 | 4.24E-13 | Negative |
| MYBL2 | ACADSB | -0.327 | 6.80E-15 | Negative |
| ETS1 | ACADSB | 0.315 | 6.82E-14 | Positive |
| CEBPB | ACADSB | -0.346 | 1.31E-16 | Negative |
| FOXP3 | ACADSB | -0.351 | 4.05E-17 | Negative |
| PML | BID | 0.521 | 7.63E-39 | Positive |
| STAT4 | BID | 0.426 | 3.27E-25 | Positive |
| BATF | BID | 0.431 | 9.01E-26 | Positive |
| PBX1 | BID | -0.364 | 2.23E-18 | Negative |
| CENPA | BID | 0.355 | 1.98E-17 | Positive |
| RUNX1 | BID | 0.426 | 3.45E-25 | Positive |
| LMNB1 | BID | 0.436 | 2.00E-26 | Positive |
| MYBL2 | BID | 0.406 | 7.79E-23 | Positive |
| CEBPB | BID | 0.530 | 1.93E-40 | Positive |
| FOXP3 | BID | 0.405 | 1.15E-22 | Positive |
| LEF1 | BID | 0.323 | 1.60E-14 | Positive |
| EZH2 | PYCR1 | 0.371 | 5.07E-19 | Positive |
| E2F1 | PYCR1 | 0.408 | 4.32E-23 | Positive |
| FOXM1 | PYCR1 | 0.633 | 9.84E-62 | Positive |
| NCAPG | PYCR1 | 0.542 | 2.09E-42 | Positive |
| CENPA | PYCR1 | 0.593 | 1.98E-52 | Positive |
| RUNX1 | PYCR1 | 0.449 | 3.93E-28 | Positive |
| LMNB1 | PYCR1 | 0.439 | 7.56E-27 | Positive |
| MYBL2 | PYCR1 | 0.638 | 7.09E-63 | Positive |
| E2F7 | PYCR1 | 0.527 | 8.76E-40 | Positive |
| SALL4 | PYCR1 | 0.462 | 6.40E-30 | Positive |
| GATA4 | PYCR1 | 0.328 | 5.63E-15 | Positive |
| LEF1 | PYCR1 | 0.337 | 8.48E-16 | Positive |
| RBP2 | SLC25A27 | 0.395 | 1.44E-21 | Positive |
| POU5F1 | SLC25A27 | 0.348 | 8.59E-17 | Positive |
| MEF2B | SLC25A27 | 0.309 | 2.22E-13 | Positive |
| GATA3 | STAR | 0.406 | 7.83E-23 | Positive |
| MYBL2 | STAR | 0.354 | 2.20E-17 | Positive |
| SPDEF | STAR | 0.508 | 9.34E-37 | Positive |
| MYB | STAR | 0.547 | 2.20E-43 | Positive |
| SPIB | STAR | 0.384 | 2.15E-20 | Positive |

Table S5. The binding sites of transcription factors in the promoter region of prognostic MTGs were predicted by JASPAR database.

| Gene | Name | Score | Relative score | Predicted sequence |
| --- | --- | --- | --- | --- |
| ACAD11 | ETS1 | 7.81931 | 0.864824426 | GCCGGATAGG |
|  | ETS1 | 6.86791 | 0.848656925 | AAAGGATATA |
|  | CEBPB | 15.4494 | 0.981882518 | GATTGCACCAC |
|  | CEBPB | 12.2369 | 0.948207895 | GGTTTCACCAT |
| ACADSB | BATF | 9.56372 | 0.876133601 | TGTGACTAAAT |
|  | BATF | 6.59189 | 0.816548478 | GATTAATCAAA |
|  | PBX1 | 12.2535 | 0.89120126 | ACATAAATCAAA |
|  | PBX1 | 11.1458 | 0.863736086 | TCATCAATAATA |
|  | MEF2B | 6.45879 | 0.834387321 | AATAATAATAAC |
|  | MEF2B | 6.1225 | 0.830421093 | GCTTTAAATACA |
|  | MYBL2 | 6.97826 | 0.78731054 | AGGCTTTAAACGTCC |
|  | MYBL2 | 6.40515 | 0.77950972 | GGACGTTTAAAGCCT |
|  | ETS1 | 7.95957 | 0.867207861 | AGCGGAAGAA |
|  | ETS1 | 6.59484 | 0.844016698 | CCAGGATGTT |
|  | CEBPB | 8.21708 | 0.906071904 | TCTTTCAAAAC |
|  | CEBPB | 6.10909 | 0.883975679 | GATTGTATAAT |
|  | FOXP3 | 9.35863 | 0.951146459 | GCAAACA |
|  | FOXP3 | 8.5041 | 0.929646506 | ACAAACA |
| BID | BATF | 10.1892 | 0.888673957 | TATGATTCAAA |
|  | BATF | 9.88366 | 0.882548478 | TTTGAATCATA |
|  | PBX1 | 9.88004 | 0.832351896 | ACATCCATCCAT |
|  | PBX1 | 8.5711 | 0.79989647 | TTACCATTCAAT |
|  | RUNX1 | 10.9642 | 0.913423479 | CATTGTGGTTG |
|  | RUNX1 | 9.24068 | 0.8687815 | ATATGAGGTAA |
|  | MYBL2 | 2.40218 | 0.725023973 | ATTCATTAACCAGTT |
|  | MYBL2 | 2.37698 | 0.724680898 | GACCGTCACCCCTTC |
|  | CEBPB | 9.39731 | 0.918443293 | GGTTACACCAC |
|  | CEBPB | 6.65668 | 0.889715612 | AATTACCTCAT |
|  | FOXP3 | 9.35863 | 0.951146459 | GCAAACA |
|  | FOXP3 | 8.13462 | 0.920350612 | GAAAACA |
|  | LEF1 | 14.7343 | 0.885472933 | AAACATCAAAAGCTT |
|  | LEF1 | 13.9894 | 0.875473847 | CAGCATCAAAGGAAA |
| PYCR1 | E2F1 | 6.67321 | 0.795969457 | TTTCCCAC |
|  | E2F1 | 6.67321 | 0.795969457 | TTTCCCCC |
|  | RUNX1 | 7.08657 | 0.812986862 | CTCTGTGGCTT |
|  | RUNX1 | 6.36407 | 0.794273 | CACTGTGGGTT |
|  | MYBL2 | 2.83179 | 0.730871486 | GCCAGTTTCCCTGCT |
|  | MYBL2 | 2.63859 | 0.728241822 | AGCAGGGAAACTGGC |
|  | E2F7 | 0.967714 | 0.726749883 | AGTTCCCGGGATAA |
|  | E2F7 | -0.920012 | 0.706016945 | AGCCCCCTCCAAAA |
|  | GATA4 | 7.78612 | 0.827153781 | GGCCTCATCTTG |
|  | GATA4 | 5.85771 | 0.787103968 | TTCCTTTTTTTT |
|  | LEF1 | 3.62421 | 0.736337563 | AGACATGCAGGGGAA |
|  | LEF1 | 3.34898 | 0.732643023 | AAGGACCCAAGGATC |
| SLC25A27 | POU5F1 | 10.2368 | 0.893847677 | TCATACAAATT |
|  | POU5F1 | 9.82388 | 0.884684582 | ATATGCAATTT |
|  | MEF2B | 12.7246 | 0.908288032 | GCTAAAAATACA |
|  | MEF2B | 8.58578 | 0.859473607 | AATAAAAATAAC |
| STAR | GATA3 | 11.3728 | 0.947947393 | AGATAAAC |
|  | GATA3 | 6.5876 | 0.872653708 | AGCTAAGA |
|  | MYBL2 | 3.23275 | 0.73632916 | CACTGTTGATCTGAT |
|  | MYBL2 | 2.68912 | 0.728929559 | TGCCGTAAGACTGAT |
|  | SPDEF | 3.05201 | 0.790516067 | GGCCGGGTGTG |
|  | SPDEF | 2.59489 | 0.784130591 | AACAGGAGCTG |
|  | MYB | 5.70928 | 0.849571512 | ATGAACTGTA |
|  | MYB | 5.65617 | 0.84844081 | TACAACTATC |
|  | SPIB | 11.4386 | 0.835721319 | CCTGTCTTCCTTTTTT |
|  | SPIB | 7.32119 | 0.777614233 | GCCCAGCTCCTGTTTT |

Table S6. Difference of immune cell infiltration in patients with ccRCC under different clinical characteristics stratification.

|  |  | Age | Gender | Grade | Stage | T stage | N stage | M stage |
| --- | --- | --- | --- | --- | --- | --- | --- | --- |
|  |  | ≤65/>65 | Male/  Female | G1-2/  G3-4 | Ⅰ-Ⅱ/  Ⅲ-Ⅳ | T1-2/  3-4 | N0/  1-X | M0/  1-X |
|  |  | 353/186 | 353/186 | 249/282 | 331/205 | 349/190 | 241/298 | 428/109 |
| B.cells.naive | t | 0.623 | 1.102 | 0.280 | NA* | 0.759 | 0.403 | 0.782 |
|  | p | 0.534 | 0.271 | 0.779 | 0.291 | 0.448 | 0.687 | 0.435 |
| B.cells.memory | t | 0.177 | NA* | NA* | 1.276 | NA* | NA* | 0.799 |
|  | p | 0.860 | 0.104 | 0.856 | 0.203 | 0.339 | 0.107 | 0.425 |
| Plasma.cells | t | NA* | 1.225 | NA* | NA* | NA* | NA* | NA* |
|  | p | 0.626 | 0.221 | 0.009 | 0.021 | 0.035 | 0.864 | 0.002 |
| T.cells.CD8 | t | 0.290 | 0.819 | NA* | NA* | 2.731 | 1.270 | 2.087 |
|  | p | 0.772 | 0.413 | <0.001 | 0.002 | 0.007 | 0.205 | 0.037 |
| T.cells.CD4.memory.resting | t | 0.538 | 1.778 | 2.745 | 1.624 | 1.610 | 1.193 | 1.008 |
|  | p | 0.591 | 0.076 | 0.006 | 0.105 | 0.108 | 0.233 | 0.314 |
| T.cells.CD4.memory.activated | t | NA* | NA* | NA* | NA* | NA* | NA* | NA* |
|  | p | 0.342 | 0.287 | 0.003 | 0.086 | 0.053 | 0.848 | 0.361 |
| T.cells.follicular.helper | t | NA* | NA* | NA* | 2.853 | 2.237 | 0.748 | 1.400 |
|  | p | 0.051 | 0.269 | 0.004 | 0.005 | 0.026 | 0.455 | 0.162 |
| T.cells.regulatory..Tregs. | t | 0.050 | 0.312 | NA* | NA* | NA* | 0.724 | NA* |
|  | p | 0.960 | 0.755 | <0.001 | <0.001 | <0.001 | 0.470 | 0.005 |
| T.cells.gamma.delta | t | NA* | NA* | NA* | NA* | NA* | 1.121 | NA* |
|  | p | 0.910 | 0.929 | 0.803 | 0.145 | 0.560 | 0.263 | 0.917 |
| NK.cells.resting | t | 0.991 | 0.897 | NA* | NA* | NA* | 1.281 | NA* |
|  | p | 0.322 | 0.370 | 0.018 | 0.044 | 0.223 | 0.201 | 0.142 |
| NK.cells.activated | t | 0.913 | 1.020 | 0.500 | NA* | 0.005 | 0.133 | 2.592 |
|  | p | 0.362 | 0.308 | 0.617 | 0.995 | 0.996 | 0.895 | 0.010 |
| Monocytes | t | NA* | 0.378 | NA* | 1.226 | 1.424 | 0.284 | NA* |
|  | p | 0.687 | 0.706 | 0.012 | 0.221 | 0.155 | 0.777 | 0.723 |
| Macrophages.M0 | t | NA | NA* | 1.892 | NA* | NA* | NA* | NA* |
|  | p | 0.560 | 0.629 | 0.059 | 0.013 | 0.009 | 0.315 | 0.171 |
| Macrophages.M1 | t | 1.918 | 1.592 | NA* | 1.751 | 1.620 | 0.863 | 0.273 |
|  | p | 0.056 | 0.112 | 0.453 | 0.081 | 0.106 | 0.389 | 0.785 |
| Macrophages.M2 | t | 0.159 | 1.518 | 1.505 | 2.776 | 2.241 | 1.440 | 1.261 |
|  | p | 0.874 | 0.130 | 0.133 | 0.006 | 0.025 | 0.151 | 0.208 |
| Dendritic.cells.resting | t | NA* | NA* | NA* | NA* | NA* | NA* | NA* |
|  | p | 0.102 | 0.494 | 0.746 | 0.156 | 0.141 | 0.484 | 0.224 |
| Dendritic.cells.activated | t | NA* | NA* | 1.148 | NA* | NA* | NA* | NA* |
|  | p | 0.937 | 0.730 | 0.252 | 0.058 | 0.044 | 0.149 | 0.717 |
| Mast.cells.resting | t | NA* | 0.400 | NA* | 3.756 | NA* | NA* | 2.080 |
|  | p | 0.013 | 0.689 | <0.001 | <0.001 | <0.001 | 0.900 | 0.038 |
| Mast.cells.activated | t | 0.651 | NA* | NA* | NA* | NA* | NA* | NA* |
|  | p | 0.515 | 0.314 | 0.619 | 0.168 | 0.076 | 0.535 | 0.875 |
| Eosinophils | t | NA* | NA* | NA* | NA* | NA* | NA* | NA* |
|  | p | 0.852 | 0.325 | 0.063 | 0.104 | 0.065 | 0.747 | 0.355 |
| Neutrophils | t | NA* | NA* | NA* | NA* | NA* | NA* | NA* |
|  | p | 0.794 | 0.835 | 0.453 | 0.910 | 0.613 | 0.104 | 0.553 |

NA, not available; *, non-parametric Mann-Whitney rank sum test.

Table S7. The clinical information of corresponding ccRCC patients.

|  | Patient 1 | Patient 2 | Patient 3 | Patient 4 | Patient 5 |
| --- | --- | --- | --- | --- | --- |
| Age (years) | 68 | 59 | 56 | 76 | 55 |
| Gender | male | male | female | male | female |
| BMI (kg/m2) | 20.62 | 28.07 | 20.20 | 23.53 | 22.89 |
| Tumor side | left | right | left | right | right |
| Tumor size (mm) | 42×38 | 50×46 | 83×110 | 45×38 | 60×50 |
| Tumor type | ccRCC | ccRCC | ccRCC | ccRCC | ccRCC |
| WHO/ISUP | 1 | 2 | 3 | 2 | 3 |
| ASA score | Ⅰ | Ⅱ | Ⅱ | Ⅲ | Ⅱ |
| Operation time (min) | 141 | 218 | 241 | 167 | 192 |
| Preoperative red blood cell count (×10^12/L) | 3.01 | 4.48 | 3.39 | 4.32 | 4.49 |
| Preoperative hemoglobin (g/L) | 104 | 134 | 104 | 143 | 126 |
| Preoperative serum urea (mmol/L) | 8.6 | 5.8 | 10.02 | 8.3 | 6.9 |
| Preoperative serum creatinine (μmol/L) | 139 | 135 | 127 | 87 | 73 |
| Preoperative serum uric acid (μmol/L) | 430 | 323 | 391 | 424 | 355 |
| Preoperative eGFR (ml/min/1.73^2) | 44.5 | 49.2 | 40.7 | 74.2 | 80.1 |
| Postoperative red blood cell count (×10^12/L) | 2.55 | 4.66 | 2.73 | 3.95 | 4.06 |
| Postoperative hemoglobin (g/L) | 87 | 137 | 84 | 135 | 113 |
| Postoperative serum urea (mmol/L) | 9.3 | 5 | 7.6 | 5.4 | 5.73 |
| Postoperative serum creatinine (μmol/L) | 165 | 143 | 111 | 72 | 91 |
| Postoperative serum uric acid (μmol/L) | 409 | 290 | 235 | 173 | 252 |
| Postoperative eGFR (ml/min/1.73^2) | 36.2 | 45.9 | 47.9 | 86.1 | 61.4 |


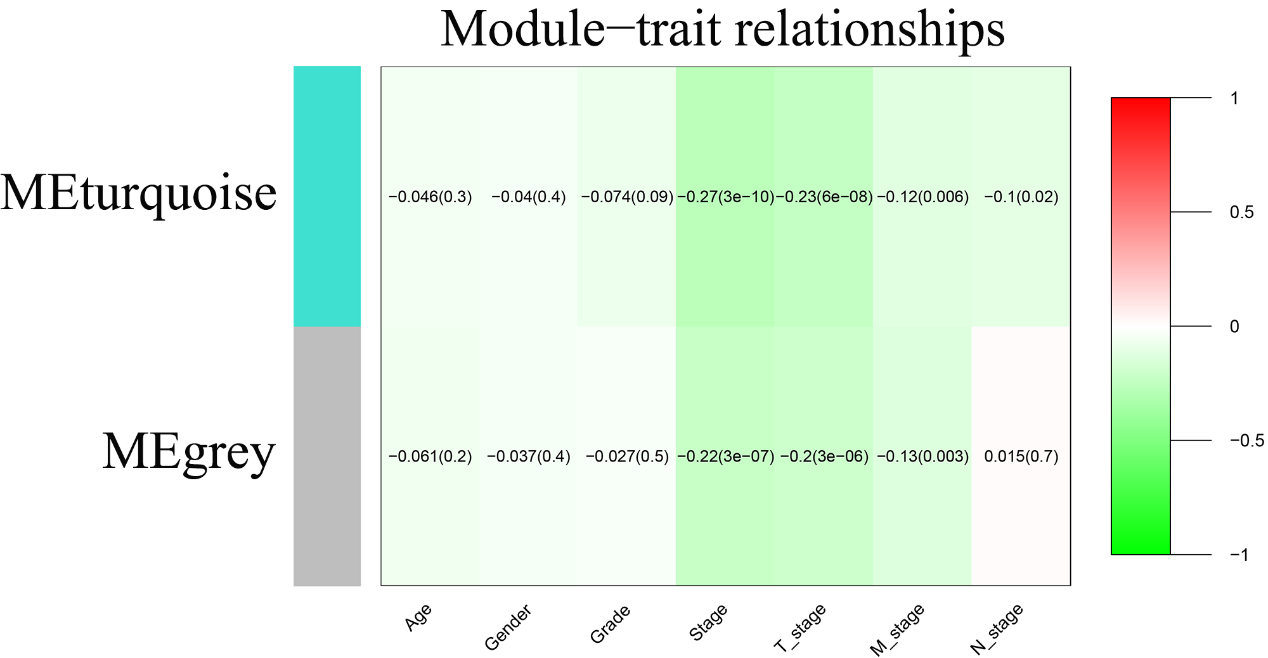


Figure S1. Relationship between gene modules and clinical variables based on WGCNA analysis. The correlation coefficients and corresponding *P* values between specific gene modules and clinical variables are shown in the boxes.


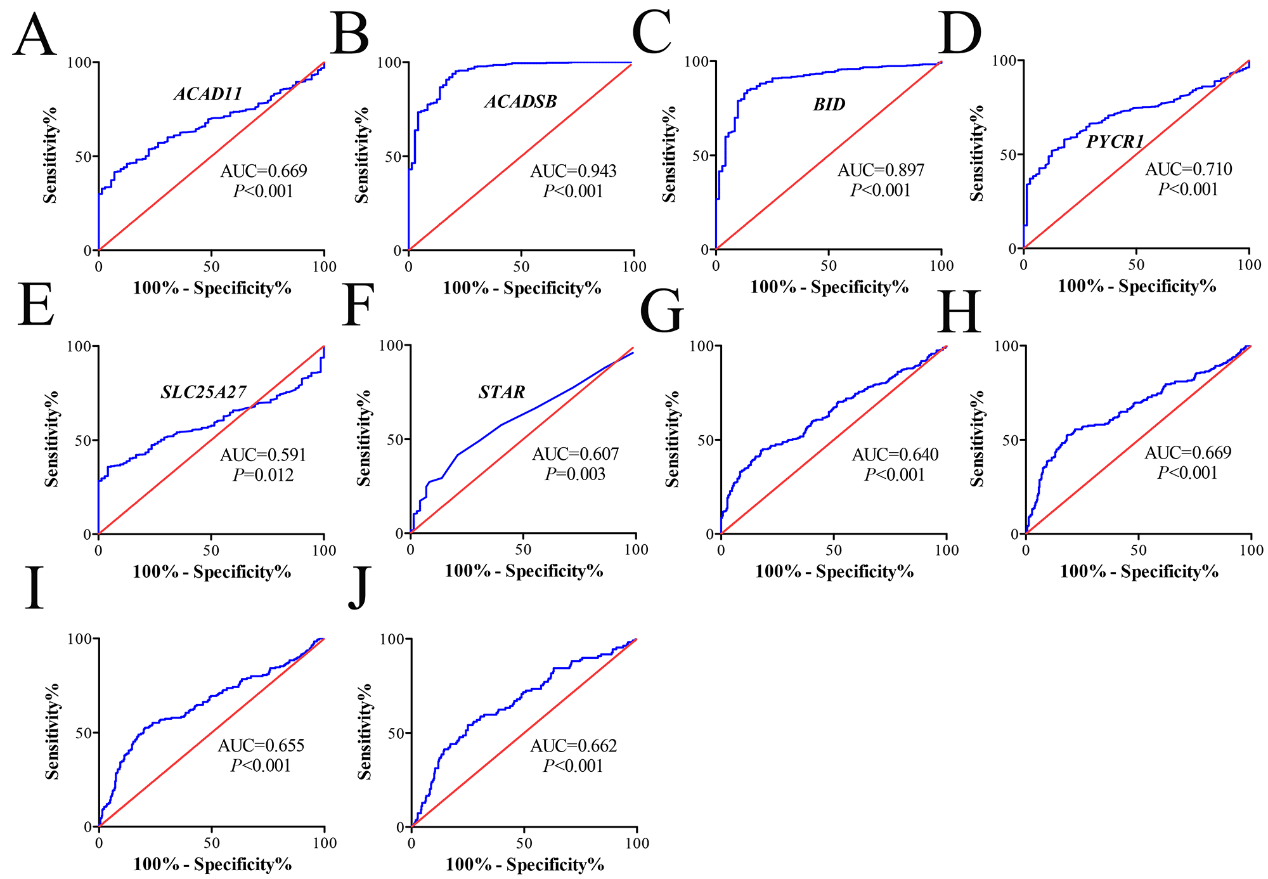


Figure S2. ROC curve analysis of prognostic MTGs and signature.

(A) The AUC of *ACAD11* in ccRCC and normal renal tissues; (B) The AUC of *ACADSB* in ccRCC and normal renal tissues; (C) The AUC of *BID* in ccRCC and normal renal tissues; (D) The AUC of *PYCR1* in ccRCC and normal renal tissues; (E) The AUC of *SLC25A27* in ccRCC and normal renal tissues; (F) The AUC of *STAR* in ccRCC and normal renal tissues; (G) The AUC of prognostic signature in tumor grade; (H) The AUC of prognostic signature in tumor stage; (I) The AUC of prognostic signature in T stage; (J) The AUC of prognostic signature in M stage.


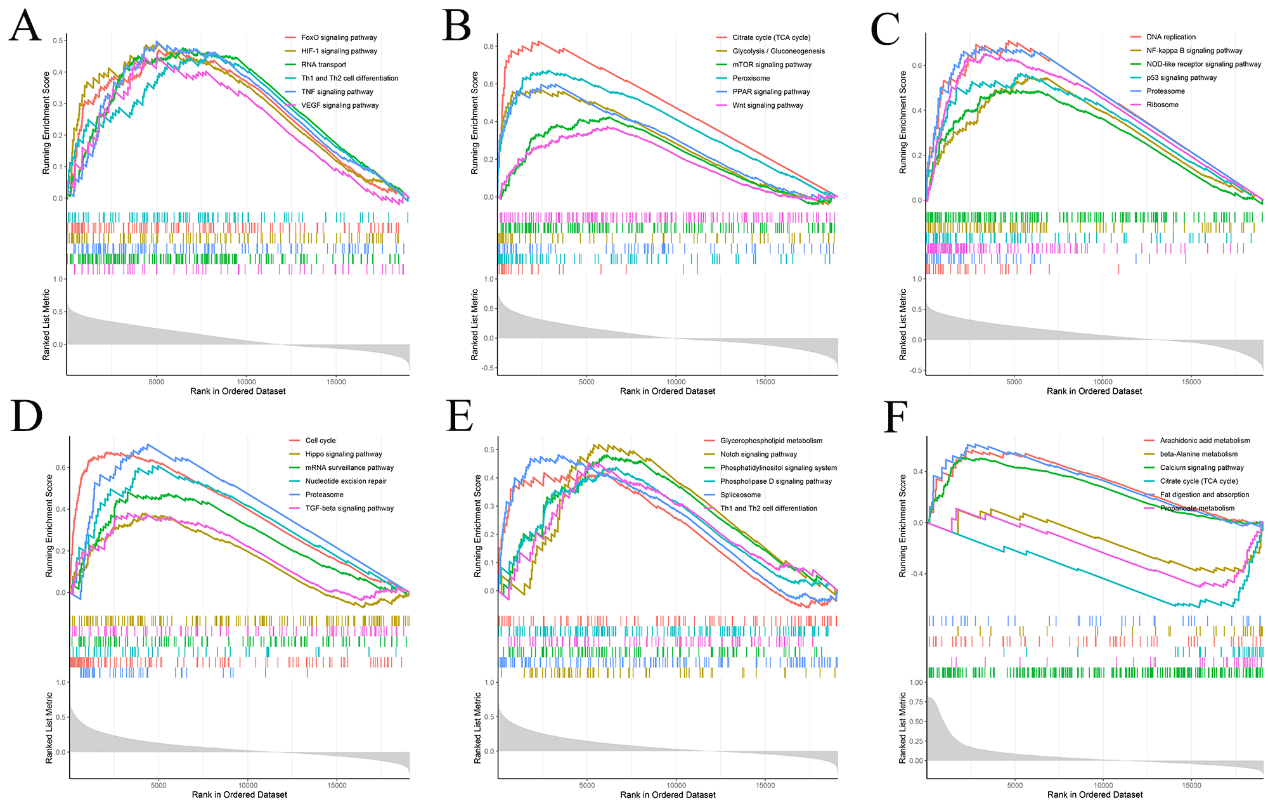


Figure S3. GSEA of prognostic MTGs.

(A-F) The results of enrichment analysis by GSEA for *ACAD11*, *ACADSB*, *BID*, *PYCR1*, *SLC25A27*, and *STAR*.


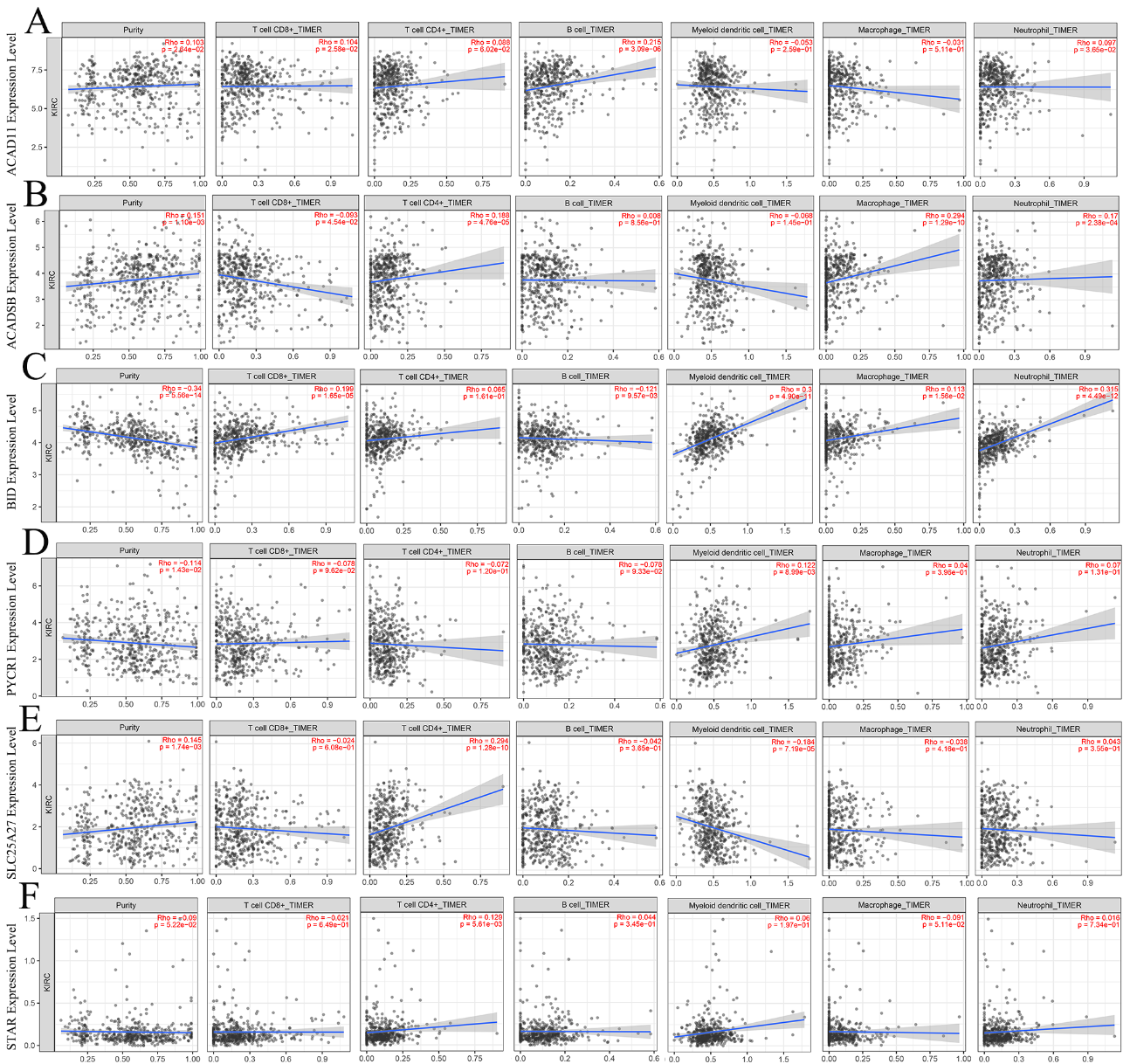


Figure S4. Correlation of prognostic MTGs with immune infiltration in ccRCC.

Results of correlation between immune infiltration level and expression of ACAD11 (A), ACADSB (B), BID (C), PYCR1 (D), SLC25A27 (E), and STAR (F) in ccRCC.


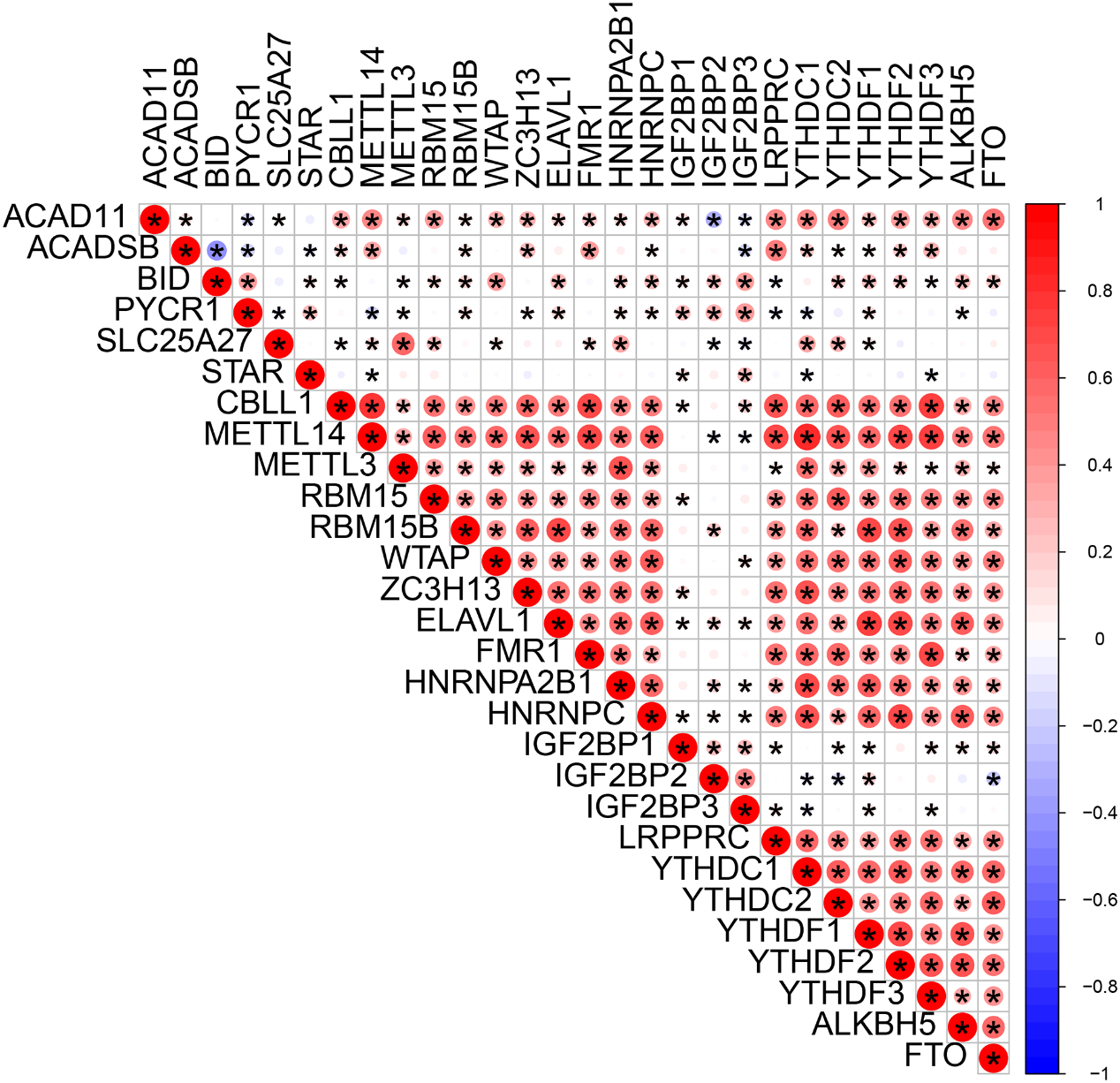


Figure S5. Correlation of prognostic MTGs with RNA modification in ccRCC.

*
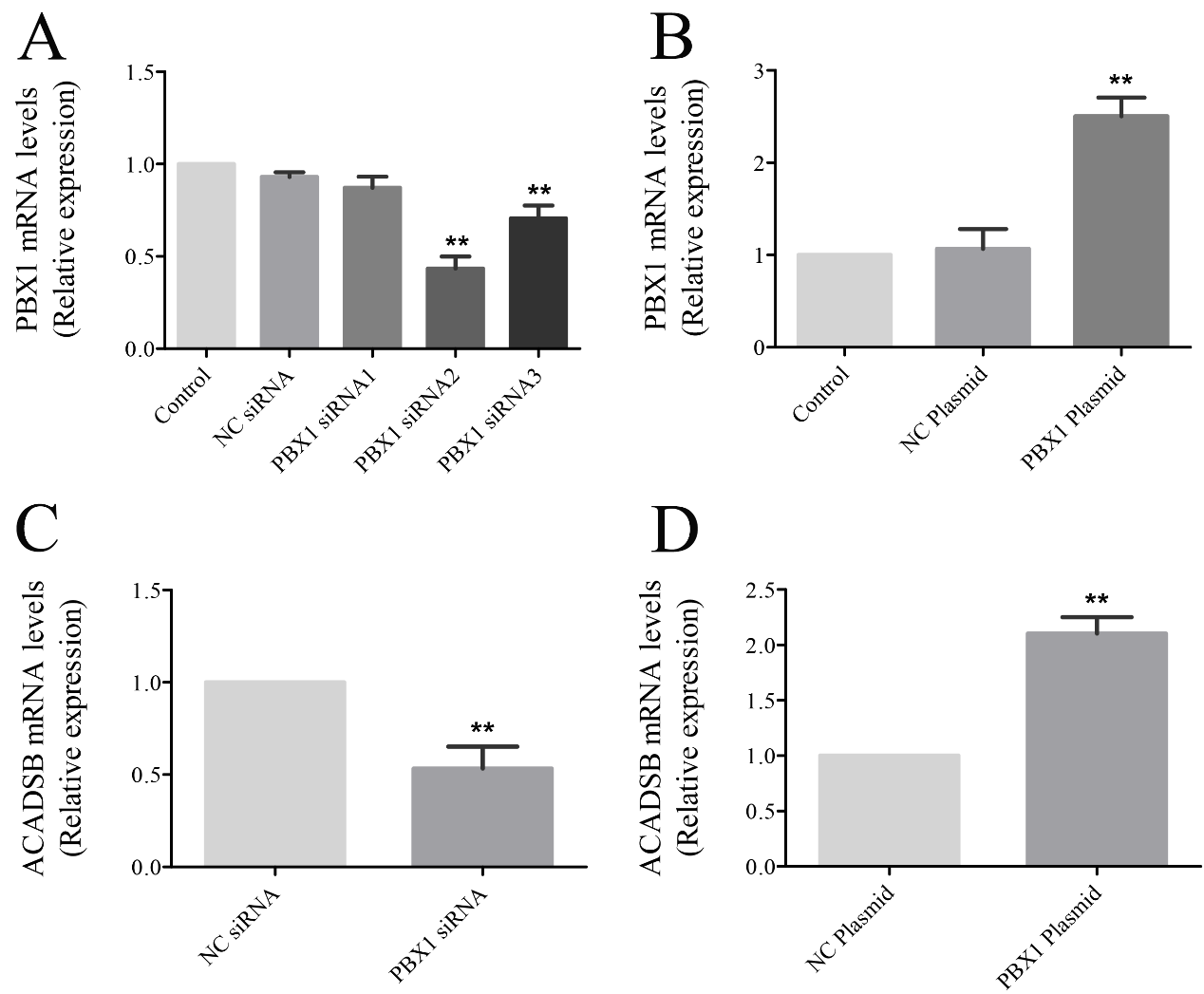
*

Figure S6. RT-qPCR results were obtained after transfection of PBX1 overexpressed plasmid and interference plasmid.

(A) Transcript levels for PBX1 were assessed following transfection of scrambled control RNA (NC siRNA) or PBX1-specific siRNA (PBX1 siRNA1-3) in OS cells; (B)

Transcript levels for PBX1 were assessed following transfection of scrambled control RNA (NC Plasmid) or PBX1 Plasmid in OS cells; (C) Transcript levels for ACADSB were assessed following transfection of scrambled control RNA (NC siRNA) or PBX1-specific siRNA (PBX1 siRNA2) in OS cells; (D) Transcript levels for ACADSB were assessed following transfection of scrambled control RNA (NC Plasmid) or PBX1 Plasmid in OS cells. ***P* < 0.01 versus NC group.


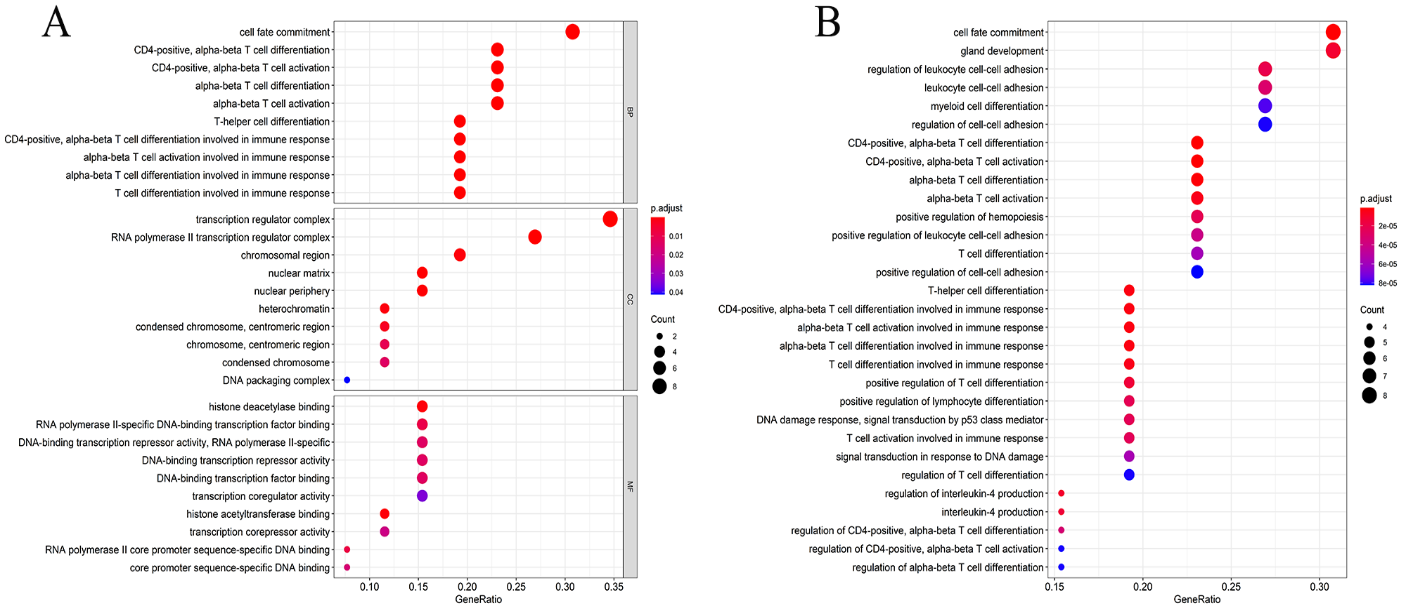


Figure S7. Functional enrichment analysis of TFs.

(A) GO enrichment analysis of TFs involved in regulation; (B) KEGG enrichment analysis of TFs involved in regulation.


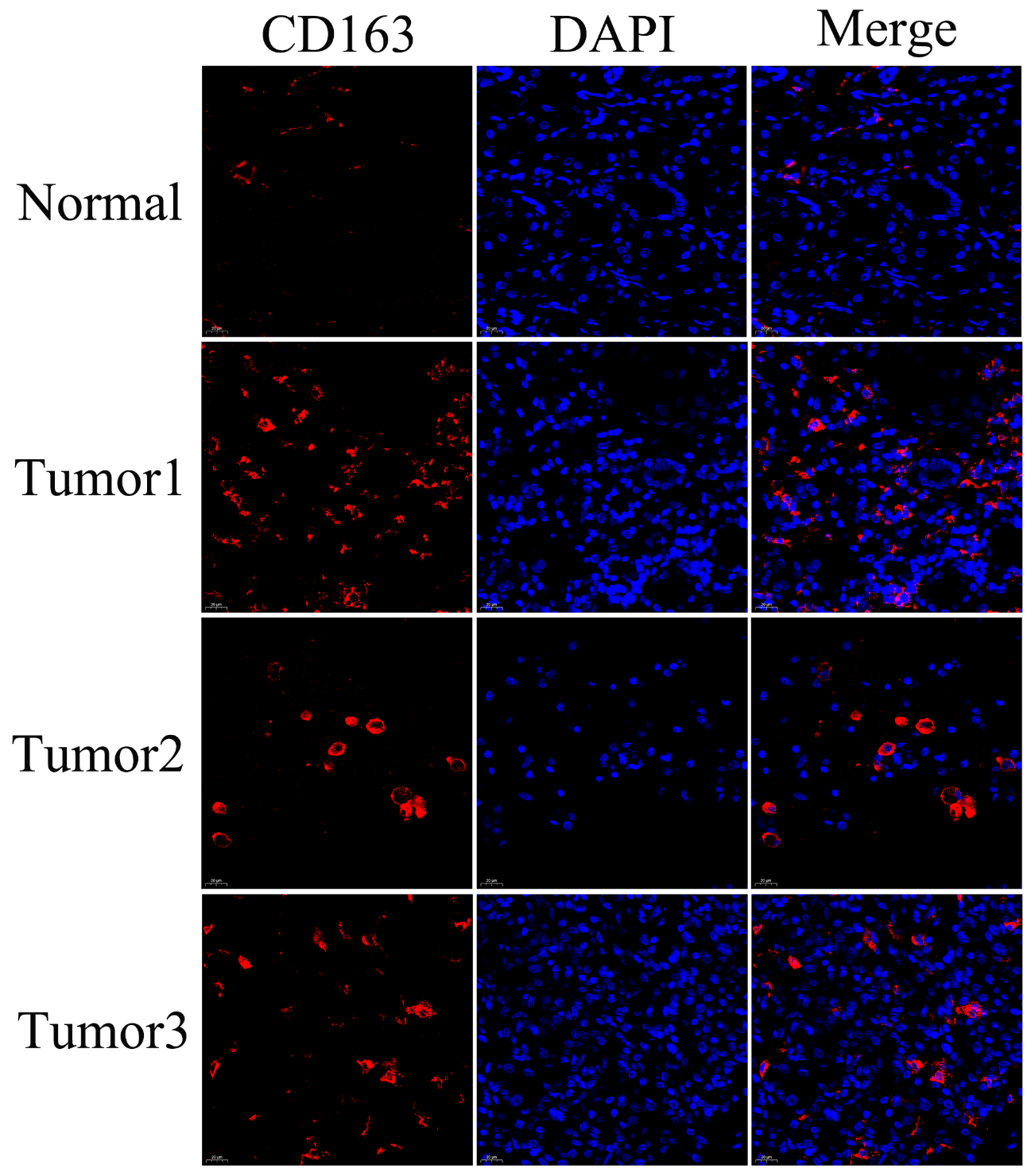


Figure S8. Immunofluorescence analysis of M2 macrophage marker CD163 in ccRCC and normal renal tissue.


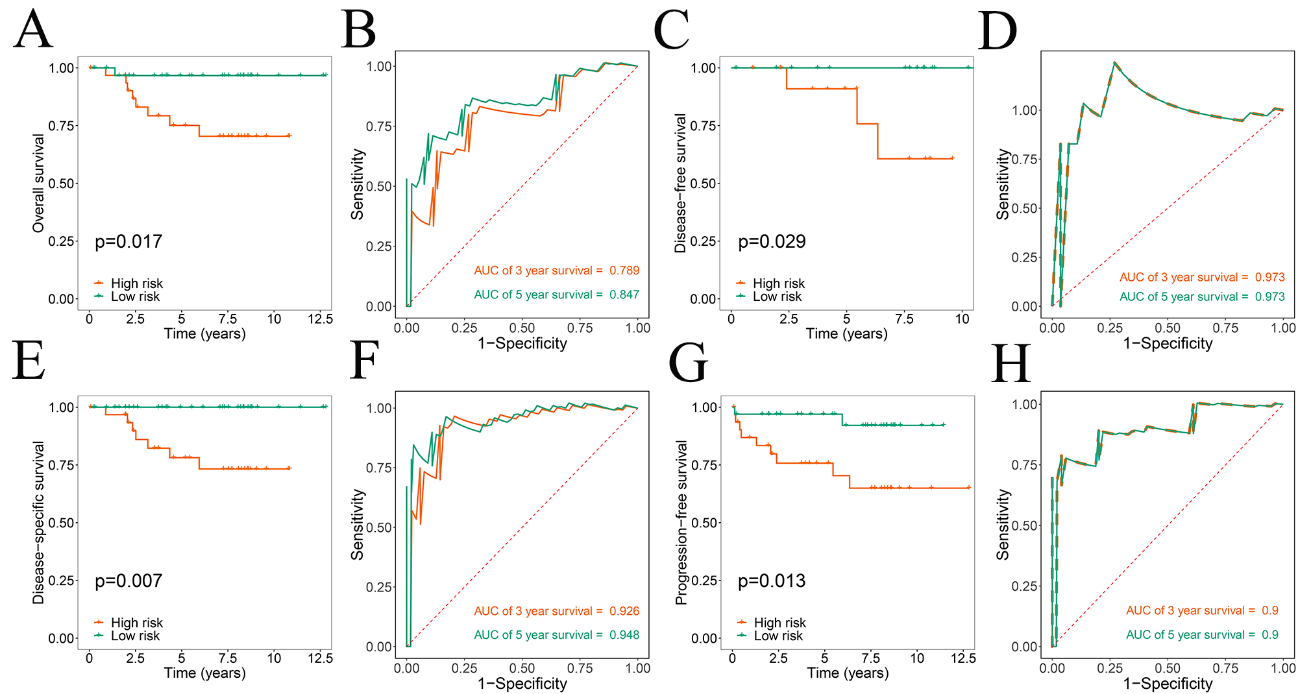


Figure S9. Prognostic potential of the MTGs-based signature in chRCC.

(A) Kaplan-Meier survival curves analysis for overall survival grouped by median risk score in the TCGA cohort; (B) Time-dependent ROC curves for overall survival in the TCGA cohort; (C) Kaplan-Meier survival curves analysis for disease-free survival grouped by median risk score in the TCGA cohort; (D) Time-dependent ROC curves for disease-free survival in the TCGA cohort; (E) Kaplan-Meier survival curves analysis for disease-specific survival grouped by median risk score in the TCGA cohort; (F) Time-dependent ROC curves for disease-specific survival in the TCGA cohort; (G) Kaplan-Meier survival curves analysis for progression-free survival grouped by median risk score in the TCGA cohort; (H) Time-dependent ROC curves for progression-free survival in the TCGA cohort.
